# Graphene-Based Biosensors: Enabling the Next Generation of Diagnostic Technologies—A Review

**DOI:** 10.3390/bios15090586

**Published:** 2025-09-06

**Authors:** John Paolo Ramoso, Manoochehr Rasekh, Wamadeva Balachandran

**Affiliations:** College of Engineering, Design and Physical Sciences, Brunel University of London, Uxbridge UB8 3PH, UK

**Keywords:** graphene, biosensors, detection mechanisms, multiplex detection, review

## Abstract

Graphene, a two-dimensional carbon material with a hexagonal lattice structure, possesses remarkable properties. Exceptional electrical conductivity, mechanical strength, and high surface area that make it a powerful platform for biosensing applications. Its sp^2^-hybridised network facilitates efficient electron mobility and enables diverse surface functionalisation through bio-interfacing. This review highlights the core detection mechanisms in graphene-based biosensors. Optical sensing techniques, such as surface plasmon resonance (SPR) and surface-enhanced Raman scattering (SERS), benefit significantly from graphene’s strong light–matter interaction, which enhances signal sensitivity. Although graphene itself lacks intrinsic piezoelectricity, its integration with piezoelectric substrates can augment the performance of piezoelectric biosensors. In electrochemical sensing, graphene-based electrodes support rapid electron transfer, enabling fast response times across a range of techniques, including impedance spectroscopy, amperometry, and voltammetry. Graphene field-effect transistors (GFETs), which leverage graphene’s high carrier mobility, offer real-time, label-free, and highly sensitive detection of biomolecules. In addition, the review also explores multiplexed detection strategies vital for point-of-care diagnostics. Graphene’s nanoscale dimensions and tunable surface chemistry facilitate both array-based configurations and the simultaneous detection of multiple biomarkers. This adaptability makes graphene an ideal material for compact, scalable, and accurate biosensor platforms. Continued advancements in graphene biofunctionalisation, sensing modalities, and integrated multiplexing are driving the development of next-generation biosensors with superior sensitivity, selectivity, and diagnostic reliability.

## 1. Introduction

Graphene is a single layer of carbon atoms arranged in a hexagonal honeycomb lattice, where each atom forms strong covalent bonds with three neighbours. It remains a focal point of nanotechnology research due to its extraordinary strength, flexibility, high surface area, and outstanding electrical and thermal conductivity [1]. These unique properties, combined with its atomic thickness and tunable electronic characteristics, have made graphene not only a versatile material for advanced applications but also a particularly promising platform for biosensing technologies.

As a two-dimensional material, graphene combines exceptional structural, mechanical, and electronic features that make it highly adaptable across scientific and engineering domains. Its thermal stability and broad operational range support applications from high-speed electronics to biomedical engineering and environmental monitoring. Crucially, advances in synthesis and processing now allow greater control over graphene’s quality and scalability, enabling its integration into compact devices. These developments have positioned graphene as a core material in biosensing, where its multifunctional properties can be directly exploited to achieve highly sensitive and selective detection [2,3].

At the atomic level, graphene consists of sp^2^-hybridised carbon atoms arranged in a hexagonal lattice, with a delocalised π-electron system extending above and below its plane. This delocalisation contributes to its exceptional in-plane mechanical strength and high charge carrier mobility, supporting superior electrical and thermal conductivities [4,5]. These properties also enable various modes of functionalisation, including π–π stacking, covalent bonding, and van der Waals interactions. Additionally, graphene’s high surface area-to-volume ratio facilitates extensive molecular interactions, a feature particularly advantageous for biosensing applications that rely on detecting subtle biochemical or environmental changes.

When integrated onto a substrate, graphene may serve as a standalone electrode, extremely responsive to external physicochemical stimuli or as the active channel in field-effect transistors (FETs), where its tunable surface potential enables label-free detection of target analytes. Moreover, graphene enhances the performance of optical biosensors. Its strong light–matter interaction and elevated refractive index improve signal transduction in surface plasmon resonance (SPR) platforms, enhancing sensitivity. In surface-enhanced Raman scattering (SERS), graphene’s unique electronic and vibrational characteristics contribute to effective signal amplification, supporting high-resolution, label-free molecular detection [6,7].

Collectively, these properties underline why graphene has emerged as a central material in the design of next-generation biosensors, offering unprecedented sensitivity, versatility, and compatibility with point-of-care (POC) technologies [8,9,10,11,12,13,14,15].

Graphene’s electrical sensitivity has led to widespread interest in its application within biosensing technologies. It serves as a highly responsive transduction layer, capable of detecting subtle changes in electrical signals induced by analyte binding. Because of these unique properties, graphene has become a focal material for developing sensitive and selective detection platforms in both biomedical and engineering contexts. When employed in biosensors, graphene is generally integrated into devices through a well-defined preparation sequence: pre-treatment, functionalisation, immobilisation, blocking, and washing. The initial pre-treatment, commonly using acetone or phosphate-buffered saline (PBS), removes contaminants and residues from the graphene surface. Functionalisation follows, where linker molecules are introduced to exploit the π-electron system and facilitate subsequent binding of bioreceptors. Once functionalised, specific bioreceptors are immobilised on the graphene surface to confer analyte selectivity [7,9].

To minimise non-specific interactions, a blocking step is performed to passivate unreacted sites, a critical factor in ensuring accuracy and reproducibility. Finally, washing with agents such as PBS or deionised water helps remove unbound molecules, reducing background noise and ensuring clearer sensor output. Together, this multistep process optimises graphene’s surface for reliable biosensing applications [16].

Graphene derivatives such as graphene oxide (GO) and reduced graphene oxide (rGO) further expand its functional potential. These materials possess abundant oxygen-containing functional groups, enabling covalent and non-covalent modifications for enhanced specificity and stability. Produced mainly through chemical or mechanical exfoliation, GO and rGO are widely employed in integrated sensing circuits, where their tunable properties allow adaptation to diverse biosensing modalities. Graphene’s exceptional optoelectronic and surface-interaction properties lend themselves to diverse detection techniques. In optical sensing, graphene enhances signal response through its strong light–matter interaction and high refractive index. In surface SPR platforms, graphene supports strong plasmonic coupling with metallic substrates, leading to increased sensitivity. In photoluminescence (PL) sensing, its tunable bandgap and fluorescence-quenching properties facilitate high-resolution detection of biomolecules [17,18,19,20].

Raman spectroscopy also benefits from graphene’s electronic and vibrational characteristics. As a signal enhancer in SERS and graphene-enhanced Raman scattering (GERS), graphene improves the visibility of weak or fluorescent molecular vibrational signals. Although graphene itself lacks intrinsic piezoelectricity, its mechanical flexibility and robustness make it valuable as a coating material in piezoelectric biosensors. In these hybrid systems, graphene amplifies sensitivity to mass variations or mechanical deformations by improving signal transduction when combined with piezoelectric substrates [21,22,23,24].

In electrochemical (EC) biosensing, graphene’s high surface area and superior electron transport characteristics significantly boost performance. It facilitates sensitive detection in methods like impedance spectroscopy, amperometry, and voltammetry. For example, graphene-based electrodes improve charge transfer rates and detection resolution [25,26,27]. The performance of these sensors depends on the input parameter being measured, such as impedance, frequency, or current, and can be further enhanced through tailored surface design, electrode architecture, and incorporation of reference elements [28,29,30]. Incorporating graphene into transistor-based sensors, particularly Graphene field-effect transistors (GFETs), further extends its application potential. In GFETs, graphene functions as a semiconducting channel where analyte binding modulates conductivity in real time, enabling label-free detection of diverse targets including DNA, proteins, and gases. Depending on the sensing configuration, GFETs may employ back gates, top gates, coplanar gates, or electrolyte gating systems, each influencing detection performance and integration complexity [5,31,32].

With the ongoing shift toward personalised medicine and decentralised diagnostics, there is increasing demand for reliable, accurate, and miniaturised biosensors for POC use. Graphene-based sensors are particularly suited to these applications due to their compact size, rapid response, high specificity, and repeatability. Multiplexed detection, achieved either through spatial sensor arrays or multi-analyte functionalisation of a single sensor platform, adds further utility, supporting simultaneous measurement of multiple biomarkers within one device [33,34].

Accordingly, this review focuses on the intrinsic properties of graphene that underpin its role in biosensor development, with emphasis on its structural and electronic characteristics, chemical reactivity, and compatibility with functionalisation techniques. We discuss the major detection approaches leveraging graphene including optical, piezoelectric, electrochemical, and transistor-based mechanisms with particular attention to multiplex detection. Finally, fabrication strategies, device integration, and application domains ranging from medical diagnostics to environmental monitoring are highlighted. As the filed continues to evolve, optimising graphene-based systems will be essential for realising their potential in next-generation detection technologies.

## 2. Graphene’s Properties Relevant to Detection Devices

Graphene’s distinctive hexagonal lattice structure underpins its exceptional electrical conductivity, mechanical strength, and surface-related properties. This section explores the interplay among these characteristics, with particular emphasis on their combined impact and relevance in emerging applications such as electronic devices, advanced composite materials, and diagnostic platforms.

### 2.1. Lattice Structure and Optical Properties

**Figure 1:**
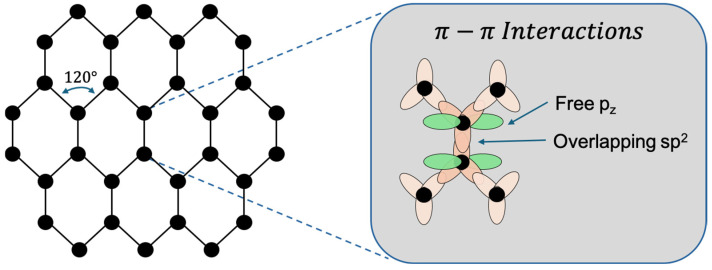
Graphene molecular lattice visualisation (designed by the authors).

Pristine graphene is composed entirely of carbon atoms arranged in a two-dimensional honeycomb-like hexagonal lattice. Each carbon atom possesses six electrons, with an electronic configuration of 1s^2^, 2s^2^, and 2p^2^. Of these, four are valence electrons, contributing to carbon’s well-known versatility in bonding behaviour [9]. While carbon atoms typically adopt a tetrahedral geometry in many compounds, in graphene they exhibit a trigonal planar configuration due to sp^2^ hybridisation. In this arrangement, the 2s, 2p_x_, and 2p_y_ orbitals combine to form three sp^2^ hybrid orbitals oriented 120° apart, which form strong covalent bonds with adjacent carbon atoms, resulting in graphene’s characteristic hexagonal structure (Figure 1) [35]. This planar architecture is not only mechanically robust but also highly efficient at distributing mechanical stress uniformly, contributing to graphene’s extraordinary strength, surpassing that of any known natural material [17]. The unhybridised 2p_z (2p_z_) orbital remains perpendicular to the plane, allowing the formation of a delocalised π-electron cloud above and below the graphene sheet. This delocalised π-system is fundamental to graphene’s exceptional electrical conductivity and also plays a role in its optical activity and emerging biocompatibility.

Graphene is widely recognised for its exceptional mechanical strength, electrical conductivity, and thermal stability. These properties make it an attractive material for reinforcing composite, enhancing catalytic processes, and improving the performance of sensing platforms. For instance, its high mechanical strength enhances the durability of composite materials, while its superior electronic mobility supports faster and more reliable signal transduction in sensors and electronic devices [6,36,37]. In addition, efficient heat dissipation enabled by graphene’s thermal conductivity is critical in high-performance applications. Collectively, these attributes have accelerated advances across diverse fields, including biomedicine, electronics, and energy storage [2,3]. Structurally, graphene consists of sp^2^-hybridised carbon atoms arranged in a hexagonal lattice, with delocalised π-electrons extending above and below the atomic plane [9]. This configuration accounts for its outstanding electron mobility and mechanical strength, which together underpin its exceptional transport and stability characteristics. As a single-atom-thick monolayer, graphene combines flexibility with structural integrity, while its high surface-to-volume ratio provides numerous active sites for chemical functionalisation. These features, coupled with excellent conductivity, render graphene highly responsive to subtle electrical variations, establishing it as a powerful platform for detecting biomolecular interactions [5].

### 2.2. Electrical Conductivity

Graphene exhibits significant potential across a broad spectrum of advanced sensor technologies due to its exceptional electrical, electrochemical, and optical properties [6]. Its high electrical conductivity originates from the delocalised π-electron system associated with its hexagonal carbon lattice structure. This configuration supports exceptionally high carrier mobility and charge capacity, facilitating rapid sensor response times and enhancing both sensitivity and signal-to-noise ratios [38,39]. In EC sensors, graphene’s large specific surface area combined with its unique electronic characteristics promotes efficient and rapid electron transfer, leading to improved sensitivity and swift analyte detection. Furthermore, graphene’s optical properties, particularly its ability to quench fluorescence, are valuable for the development of fluorescence-based sensors. Fluorescence quenching, the process by which a molecule’s fluorescence is diminished through photochemical interactions, enables precise detection of target analytes in a variety of sensing platforms [35,40].

Beyond electronic transport, graphene’s hexagonal lattice also supports efficient phonon propagation and quantised vibrational energy modes due to the strength of its carbon–carbon bonds [41]. These phonons interact with charge carriers, influencing electron mobility and scattering behaviour [1,6]. The unique phonon (lattice vibration) dispersion characteristics inherent to graphene’s two-dimensional crystalline structure contribute significantly to its outstanding electrical and thermal conductivity.

### 2.3. Flexibility and Mechanical Strength

Graphene exhibits exceptional mechanical strength while remaining remarkably lightweight and flexible, making it highly suitable for a broad range of structural and sensing applications. Its atomic structure imparts a high tensile strength and low density, along with a large specific surface area. Notably, graphene possesses a Young’s modulus of up to 1100 GPa and a specific surface area of approximately 2630 m^2^.g^−1^ [17]. These properties enable graphene to be effectively transferred onto and integrated with a wide variety of substrates, including flexible and irregular surfaces, which is particularly advantageous for wearable sensors and devices designed to conform to complex geometries [23,42].

The unique combination of flexibility and mechanical robustness positions graphene as a highly promising candidate for use in piezoelectric and piezo-resistive devices. For instance, graphene-based piezo-resistive pressure sensors provide low-cost, flexible solutions with high sensitivity. In such systems, applied pressure alters the contact area between the graphene-based conductive layer and the electrodes, leading to measurable changes in electrical resistance. These resistance variations reflect deformation-induced modifications in the conductive pathways within the sensing material, allowing for precise and repeatable pressure detection [43].

### 2.4. Surface Area and Chemical Reactivity

Owing to its two-dimensional, single-atom-thick structure, graphene exhibits an exceptionally high surface area relative to its volume [35]. This expansive surface area, combined with its unique chemical and electrical properties, significantly enhances graphene’s performance as a transducer in sensing systems. The delocalised π-electron system across its surface enables strong interactions with various functional groups such as hydroxyl (–OH), carbonyl (–O), and fluorine (–F), making it highly responsive to chemical modifications. These interactions facilitate both the adsorption of biomolecules and the modulation of graphene’s electronic properties, which are critical for developing sensitive and selective sensing platforms [44,45].

Although pristine graphene is intrinsically chemically inert due to its extended π-conjugated structure, which stabilises the carbon lattice and limits reactivity, chemically processed or synthesised graphene typically exhibits enhanced reactivity. This increased chemical activity arises from structural defects such as edges, vacancies, and residual impurities. In particular, oxidised carbon atoms located at edge sites or within disrupted lattice regions act as active centres for chemical reactions. These defect-rich areas often contain oxygen-bearing functional groups, which not only facilitate further chemical functionalisation but also enhance graphene’s suitability for sensing and detection applications [35,46,47].

### 2.5. Graphene Substrates in Biosensor Design and Application

#### 2.5.1. Pristine Graphene

Pristine graphene is a single-atom-thick sheet of sp^2^-bonded carbon atoms, exhibiting exceptional electrical conductivity, high surface area, and robust mechanical strength [4]. Its continuous π-conjugated lattice enables ultrafast electron transfer, allowing biochemical recognition events to be rapidly transduced into measurable signals [2]. The atomically thin planar structure also facilitates strong π–π interactions with biomolecules, providing a platform for non-covalent adsorption.

In addition, the chemical stability and low defect density of pristine graphene minimise background interference, contributing to high sensitivity in biosensing. However, the absence of intrinsic functional groups limits direct immobilisation of bioreceptors, necessitating the use of chemical or hybrid functionalisation strategies to achieve stable and selective attachment.

#### 2.5.2. Graphene Oxide (GO)

GO is a highly oxidised form of graphene containing abundant oxygenated groups, including hydroxyl (-OH), carbonyl (C=O), carboxyl (-COOH), epoxy (-C–O–C), and alkoxy (C–O) functionalities, which impart hydrophilicity and excellent dispersibility in aqueous media [37]. These functional groups provide numerous sites for covalent and non-covalent immobilisation of biomolecules, and GO’s strong fluorescence-quenching properties makes it particularly suitable for fluorescence resonance energy transfer (FRET)-based biosensors. The oxidation process disrupts the sp^2^ network, introduces structural defects, and produces characteristic wrinkles in the layers, increasing interlayer spacing [9].

GO has been widely employed in DNA sensors, immunosensors, and aptamer-based detection. Its biocompatibility and ability to form three-dimensional porous structures offer additional advantages for biosensing. However, conventional chemical synthesis often relies on strong acids and harsh oxidants, raising environmental concerns and highlighting the need for milder methods that preserve GO’s structural integrity. Its main limitation remains the disrupted sp^2^ network, which significantly reduces electrical conductivity and restricts its utility in electrochemical sensing [6].

#### 2.5.3. Reduced Graphene Oxide (rGO)

rGO is produced by partially removing oxygen-containing groups from GO through chemical, thermal, or electrochemical reduction [4]. This partial deoxygenation restores segments of the sp^2^-hybridised carbon network, improving electrical conductivity while retaining some oxygen functionalities and structural defects.

The resulting material combines two advantageous characteristics: the recovered conjugated lattice facilitates efficient electron transfer, while the remaining oxygen groups provide anchoring sites for biomolecule immobilisation. Compared with GO, rGO offers enhanced conductivity while maintaining reasonable functionalisation capacity [39]. Its defect-rich structure also supports the incorporation of nanoparticles, enzymes, proteins, and nucleic acids, enabling the creation of hybrid nanocomposites with improved sensing performance [43]. However, variability in reduction methods can produce rGO with inconsistent conductivity and surface chemistry, posing challenges for reproducibility in biosensor applications.

#### 2.5.4. Graphene Quantum Dots (GQDs)

GQDs are nanoscale fragments of graphene, typically below 100 nm, exhibiting strong quantum confinement and pronounced edge effects [9,25]. These structural features impart intrinsic PL, a property absent in bulk graphene, and allow the fluorescence to be tuned via size and surface chemistry control [35]. The abundance of edge sites provides reactive domains for functionalisation and facilitates electron transfer during sensing events.

Their small size and oxygen-rich edges also enhance water solubility and biocompatibility, making GQDs well-suited for biological applications. Beyond their optical properties, GQDs have attracted attention as biocompatible signal amplification agents, with catalytic activity and the capacity to interact with multiple biomolecules simultaneously, serving as both electro-catalysts and fluorescent labels [22]. Despite these advantages, challenges such as aggregation-induced quenching and difficulties in achieving uniform size distributions limit their widespread application in reliable biosensor design.

#### 2.5.5. Other Graphene Derivatives

Beyond the commonly studied forms, several other graphene-based structures have demonstrated potential in biosensing applications. Graphene nanoribbons (GNRs) possess tunable bandgaps and edge-rich structures that enhance semiconducting behaviour, making them suitable for nanoscale FET sensors [17]. Carbon nanotubes (CNTs), which can be viewed as rolled-up graphene sheets, offer remarkable electronic and mechanical properties that are highly advantageous for sensitive electrochemical sensing [38,44]. Graphene nanowalls (GNWs) are vertically oriented, self-organised nanosheets with a high density of sharp, exposed edges, providing enhanced surface reactivity.

Three-dimensional graphene architectures, such as foams and aerogels, combine high porosity with interconnected conductive pathways, enabling efficient biomolecule loading and signal amplification [25]. Graphene composites, formed by integrating graphene with metals, oxides, or polymers, further expand the functional landscape by introducing catalytic activity, plasmonic effects, or improved stability [9,12]. Although these derivatives broaden the design toolbox for biosensing, practical implementation is often limited by fabrication complexity, reproducibility challenges, and structural stability under operational conditions.

## 3. Graphene Biofunctionalisation

Graphene’s intrinsic properties provide substantial potential for the development of advanced detection devices. However, to fully exploit these capabilities, it is essential to tailor the surface characteristics of graphene for specific applications [31]. This process involves deliberate modifications to the graphene lattice and surface chemistry to enhance its sensitivity and selectivity toward target analytes. The initial step in biofunctionalisation involves ensuring that the graphene surface is clean and free from contaminants that could alter its electronic properties, such as unintended doping or structural defects [48,49]. Common cleaning methods include rinsing with deionised water or PBS [50]. Alternatively, more rigorous procedures involve sequential washing with acetone followed by immersion in ethyl acetate to remove organic residues [51]. Following surface preparation, graphene is functionalised by introducing specific chemical groups or linker molecules onto its surface, thereby enabling selective interaction with target analytes. After functionalisation, a bioreceptor such as an antibody, aptamer, or enzyme with high specificity for the intended analyte is immobilised onto the graphene surface. To minimise non-specific adsorption that could compromise detection accuracy, the remaining unfunctionalised regions are blocked using suitable blocking agents (e.g., bovine serum albumin or polyethylene glycol). A final rinse with PBS or another compatible washing solution is then performed to remove unbound biomolecules and residual reagents. The success of biofunctionalisation is typically confirmed using optical or spectroscopic techniques such as Raman spectroscopy, X-ray photoelectron spectroscopy (XPS), or fluorescence imaging, which can verify the presence and uniformity of the functional layers [52,53,54].

### 3.1. Functionalisation

Graphene can be functionalised using two primary strategies: covalent and non-covalent functionalisation. Both approaches serve to alter the surface chemistry and electronic properties of graphene by introducing functional groups, thereby improving its compatibility with target analytes and enabling the immobilisation of biomolecules [48,55]. Covalent functionalisation involves the formation of covalent bonds between the graphene lattice and organic molecules or functional moieties. This method allows precise control over the chemical environment of the graphene surface and can also improve its mechanical stability. A common technique employs 1-ethyl-3-(3-dimethylaminopropyl) carbodiimide hydrochloride/N-hydroxy succinimide (EDC/NHS) chemistry to activate carboxyl (–COOH) groups, facilitating covalent attachment of biomolecules such as antibodies [56]. Another widely used linker is 1-pyrenebutyric acid N-hydroxysuccinimide ester (PBASE), which enables covalent immobilisation of bioreceptors through NHS-ester-mediated crosslinking reactions. Numerous covalent strategies are available, each selected based on the nature of the graphene substrate, the biomolecule to be immobilised, and the intended sensing or diagnostic application [56,57,58,59].

In contrast, non-covalent functionalisation relies on physical interactions such as π–π stacking, hydrophobic interactions, and van der Waals forces to attach foreign molecules to the graphene surface [21,45,60]. A key advantage of this method is the preservation of graphene’s intrinsic electronic properties, as the sp^2^-hybridised carbon network remains undisturbed. For example, non-covalent interaction with aromatic compounds, polymers, or surfactants are capable of forming stable composites via van der Waals or π–π interactions. Additional methods include physisorption or the direct adsorption of biomolecules onto the graphene surface without prior modification. These techniques are generally simpler and less destructive, making them suitable for applications where maintaining graphene’s pristine electrical characteristics is crucial. However, the specificity and stability of biomolecule attachment in non-covalent approaches can be less reliable compared to covalent strategies. Various strategies have been developed for the biofunctionalisation of graphene to facilitate effective and stable bioreceptor attachment. These include covalent and non-covalent modifications, self-assembled monolayers (SAMs), polymer coatings, and aptamer-assisted functionalisation. Each method offers distinct advantages with respect to stability, biocompatibility, and the preservation of graphene’s intrinsic electronic properties [54,57,61,62,63]. A comparative overview of these functionalisation approaches along with commonly employed blocking agents and receptor immobilisation techniques is summarised in Table 1.

### 3.2. Immobilisation of Bioreceptors

Bioreceptors can be immobilised onto functionalised graphene through two primary strategies: direct immobilisation and linker-mediated immobilisation. In the direct approach, the bioreceptor binds directly to the graphene surface, whereas in the linker-mediated approach, an intermediate molecule facilitates the attachment between the bioreceptor and the graphene. In both cases, an incubation period is typically required to ensure stable and effective binding of the bioreceptor or linker–bioreceptor complex to the surface [52,64,71].

Direct immobilisation involves the attachment of bioreceptors to the graphene surface without the use of intermediary linkers. This can occur via either non-covalent or covalent interactions. Non-covalent approaches rely on physical forces such as π–π stacking, van der Waals interactions, or hydrophobic forces to adsorb the bioreceptor onto the surface. These methods are advantageous when preserving the pristine electronic properties of graphene is essential. On the other hand, covalent direct immobilisation takes advantage of intrinsic or introduced functional groups on the graphene surface particularly in oxidised forms such as GO, which presents a high density of reactive oxygen-containing groups (e.g., epoxide, carboxyl, and hydroxyl). These groups can form stable covalent bonds with bioreceptors, providing robust and long-lasting attachment [68,70,77].

In contrast, linker-mediated immobilisation involves the use of bifunctional molecules that serve as molecular bridges between the bioreceptor and the graphene surface. Commonly employed linkers include pyrene derivatives such as PBASE, carbodiimide compounds such as EDC/NHS (1-ethyl-3-(3-dimethylaminopropyl) carbodiimide/N-hydroxysuccinimide), silane-based linkers like (3-aminopropyl) triethoxysilane (APTES), and affinity-based systems such as the avidin–biotin interaction. While this strategy introduces additional preparation steps, it often provides greater stability and allows for more controlled and oriented bioreceptor attachment. To further enhance selectivity and minimise non-specific binding, optimisation steps such as quenching unreacted succinimidyl esters using ethanolamine are commonly applied [65,67,73,78].

A comparative study by Jahromi et al. [52] investigated the performance of GFETs functionalised with aptamers via both direct and linker-mediated immobilisation strategies. The results indicated that while both methods yielded comparable carrier mobility and sensitivity enhancements, direct immobilisation offered a more streamlined and less complex functionalisation route. This suggests that although linker-mediated immobilisation provides improved control and stability, further comparative studies particularly involving parallel biosensor fabrication and long-term performance analysis are essential to fully elucidate the trade-offs between performance, stability, and fabrication complexity associated with each method.

### 3.3. Blocking

The final step in the biofunctionalisation process is blocking, which plays a critical role in preventing non-specific interactions and enhancing the overall specificity and performance of graphene-based biosensors. Following the successful immobilisation of biorecognition elements such as functional groups, linkers, and bioreceptors, residual unmodified areas on the graphene surface remain vulnerable to the non-specific adsorption of interfering molecules. To address this, a blocking step is introduced to passivate these exposed regions, including unreacted linker sites and free π-bonds. Bovine serum albumin (BSA) is one of the most commonly used blocking agents, owing to its effectiveness in covering non-functionalised surface areas and preventing non-specific binding. Its widespread use is attributed to its stability, low cost, and biocompatibility. In addition to BSA, several proprietary and synthetic blocking agents have been developed to further improve passivation efficiency. These include SuperBlock™ [65], which provides rapid and uniform surface coverage, and surfactants such as Tween-20, which reduce surface tension and inhibit non-specific protein adsorption [64,73,76].

**Figure 2:**
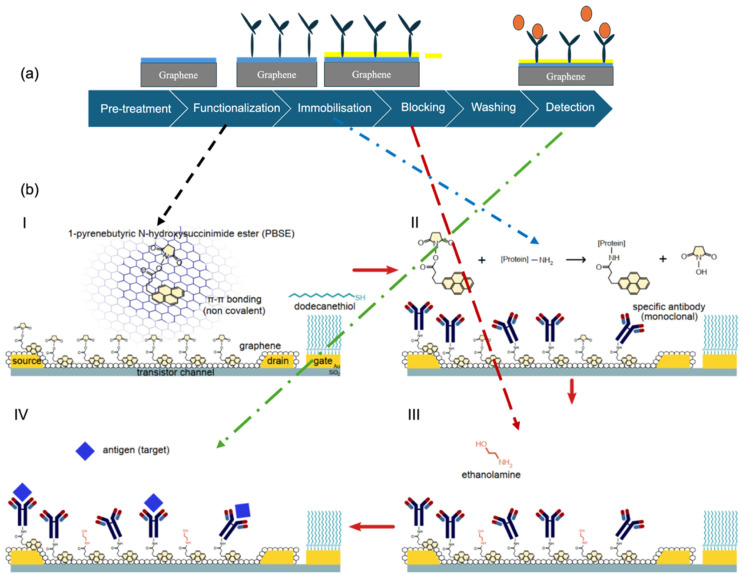
Overview of the biofunctionalisation process for graphene-based biosensors. (**a**) Schematic workflow showing key steps: pre-treatment, functionalisation, immobilisation, blocking, washing, and detection (designed by the authors). (**b**) Pictorial representation of representative molecular interactions at each step: (I) Surface modification with PBSE, (II) covalent antibody immobilization, (III) blocking with ethanolamine, and (IV) biorecognition of the MMP-9 target (Reprinted with permission from Ref. [49]. Copyright 2019, Elsevier).

These blocking agents collectively help ensure that the sensor surface responds selectively to the target analyte by minimising background noise and false positives due to non-specific adsorption. Figure 2a illustrates the modular workflow of graphene biofunctionalisation, beginning with surface activation and proceeding through linker-mediated functionalisation, bioreceptor immobilisation, and final surface passivation. Figure 2b provides a molecular perspective on key interface interactions, such as π–π stacking of PBASE on the graphene lattice and subsequent coupling of bioreceptors, offering both procedural and structural insight into surface engineering strategies for biosensing applications [49].

## 4. Detection Mechanisms

Graphene-based biosensors utilise a variety of detection mechanisms that take advantage of graphene’s unique physical, chemical, and optical properties. These mechanisms include optical, piezoelectric, electrochemical, and transistor-based approaches, each tailored for the detection of specific analytes and adaptable to a wide range of biomedical, environmental, and industrial applications. A comprehensive understanding of these detection strategies is essential to optimise sensor performance, enable multiplex detection, and expand their practical utility across diverse domains [15,79,80].

### 4.1. Optical Biosensors

Graphene-based optical biosensors have garnered significant attention due to graphene’s remarkable optical properties, high surface-to-volume ratio, and strong affinity for biomolecular interactions. These attributes enhance the sensitivity and precision of optical detection techniques, including SPR, PL, and SERS. By facilitating efficient signal transduction and amplification, graphene enables the real-time and label-free detection of a broad range of biological targets, making optical biosensors a powerful platform for diagnostics and monitoring applications [81,82].

#### 4.1.1. Surface Plasmon Resonance (SPR)

SPR is a highly sensitive, label-free optical technique that exploits the resonant oscillation of conduction electrons at the interface between a metal (typically gold) and a dielectric material upon exposure to incident light. When biomolecules bind to the sensor surface, they alter the local refractive index, resulting in a measurable shift in the resonance angle. This shift directly correlates with analyte-binding events and can be used to monitor molecular interactions in real time. Graphene significantly enhances the performance of SPR biosensors due to its large surface area, which allows for the dense immobilisation of bioreceptors, as well as its excellent electrical conductivity and optical transparency. These properties promote strong plasmonic coupling and result in sharper, more distinct resonance shifts. Consequently, the integration of graphene into SPR platforms improves both the sensitivity and stability of the biosensor, enabling more accurate and reliable detection [6,10,83].

**Figure 3:**
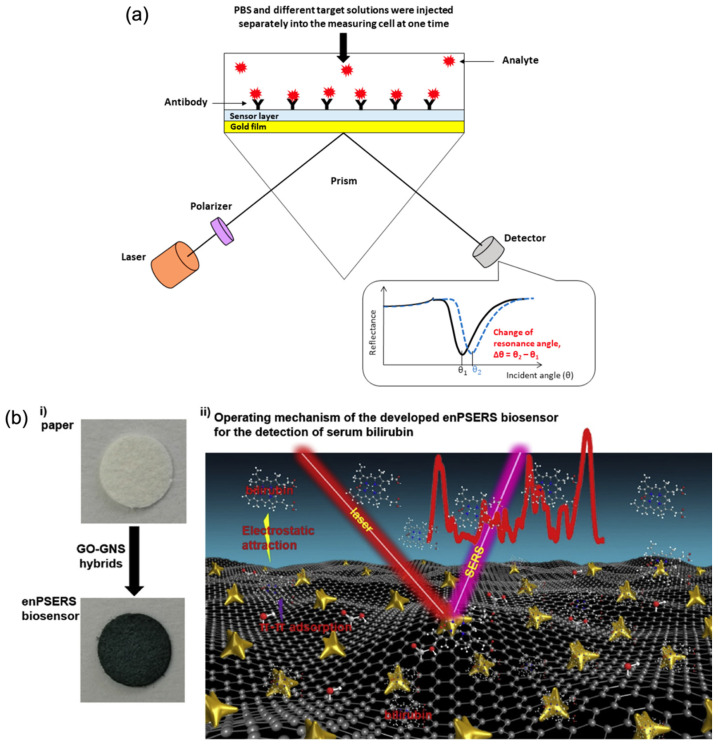
Graphene-based optical biosensing mechanisms. (**a**) SPR sensor with graphene-enhanced interface [71] and (**b**) SERS detection platform using graphene-assisted signal enhancement: (i) preparation process of the enPSERS biosensor, and (ii) operating principle for label-free SERS detection (Reprinted with permission from Ref. [87]. Copyright 2019, Elsevier).

An exemplary study by Omar et al. [71] demonstrated the effectiveness of an SPR biosensor incorporating a reduced graphene oxide–polyamidoamine (rGO–PAMAM) nanocomposite for the detection of dengue virus (DENV) 2 E-proteins. The sensor achieved a high sensitivity, with a detection limit ranging from 0.08 to 0.5 pM and a sensitivity value of 0.2576°/pM, while maintaining a strong correlation coefficient (R^2^ = 0.92). In addition to its sensitivity, the biosensor exhibited excellent selectivity, successfully distinguishing DENV 2 E-proteins from closely related DENV 1 and Zika virus E-proteins, demonstrating its potential for accurate and specific viral detection. Figure 3a presents a schematic overview of a graphene-integrated SPR biosensor. In this configuration, graphene acts as both a biorecognition platform and an optical enhancer. Its high refractive index, π-conjugated surface, and capacity for functionalisation contribute to a stronger evanescent field interaction, thereby increasing sensitivity and enabling label-free detection of low-abundance analytes.

#### 4.1.2. Photoluminescence (PL)

PL-based biosensors harness the fluorescent properties of GQDs and GO, which exhibit tunable emission characteristics depending on their size, surface chemistry, and excitation wavelength. Upon excitation with light, these materials emit PL, which can be either quenched or enhanced upon interaction with specific target analytes. This modulation in fluorescence intensity serves as a highly sensitive indicator of biomolecular interactions [18].

Graphene and its derivatives, particularly GO, are known for their strong fluorescence-quenching ability, primarily due to π–π interactions and efficient energy or electron transfer mechanisms enabled by their unique electronic structures. This makes them ideal platforms for designing PL biosensors capable of detecting trace levels of analytes [10,20].

A notable example is the work by Park and Seo [84], who developed an integrated microfluidic device for detecting trace metal ions using a graphene–oxide quantum dot (GOQD) array. The system utilised DNA aptamers specific to As^3+^, Cd^2+^, and Pb^2+^, each immobilised on separate GOQD array chips. The biosensor achieved excellent sensitivity, with detection limits of 5.03 nM (As^3+^), 41.1 nM (Cd^2+^), and 4.44 nM (Pb^2+^), demonstrating its potential for environmental monitoring of heavy metal contamination. In another application, GQDs were employed for the early detection of prostate cancer [85]. By integrating gold nanoparticles (AuNPs) with the GQD-based biosensing electrode, the platform achieved an ultra-low detection limit of 211 fM, with a linear detection range spanning from 1 μM to 100 fM and a rapid response time of 5 min. This highlights the potential of PL-based graphene biosensors for clinical diagnostics and early disease detection.

#### 4.1.3. Raman Spectroscopy-Based Biosensors

Graphene significantly enhances the performance of SERS biosensors by serving as a substrate that amplifies the Raman signals of target molecules [22]. SERS, a vibrational spectroscopy technique, detects the inelastic scattering of light, producing a molecular fingerprint that reveals structural and compositional information about analytes. However, conventional Raman signals are inherently weak, and fluorescence interference often hampers the detection of low-concentration organic molecules [6,86].

To overcome this limitation, graphene is commonly combined with metallic nanostructures (e.g., gold or silver nanoparticles), which enhance local electromagnetic fields and quench interfering fluorescence signals. Graphene contributes to SERS performance by offering uniform adsorption sites, excellent biocompatibility, and a high surface area, all of which facilitate efficient biomolecule immobilisation and improve signal uniformity and reproducibility [20,21,35].

In a representative study, Pan et al. [87] developed a hybrid graphene oxide–gold nanostar (GO–GNS) biosensor on a filter-paper substrate for the detection of serum bilirubin, relevant to jaundice diagnostics. This enhanced plasmonic SERS (enPSERS) platform leveraged the fluorescence-quenching capacity of GO and the plasmonic enhancement of gold nanostars to improve detection sensitivity. As shown in Figure 3b, the combination of graphene and metallic nanostructures enabled strong Raman signal amplification and stable biomolecule interaction. The biosensor exhibited dual linear response ranges from 5.0 to 150 μM and 150 to 500 μM, with a detection limit as low as 0.436 μM.

In another study, Srivastava et al. [88] demonstrated a metal-free SERS platform using GO as the active substrate for the detection of the industrial dye Rhodamine B (RhB). The GO substrate provided robust SERS enhancement, enabling detection in the micromolar range, three orders of magnitude more sensitive than conventional Raman methods.

A linear relationship was observed between SERS intensity and RhB concentration across a range from 10^−3^ M to 10^−6^ M, showcasing the feasibility of replacing traditional noble metal-based SERS substrates with graphene-based alternatives. These studies highlight the dual role of graphene in enhancing electromagnetic field interactions when paired with metallic nanostructures, and its intrinsic capacity as a fluorescence quencher and biocompatible matrix in standalone configurations.

### 4.2. Piezoelectric Biosensors

Although graphene is not inherently piezoelectric, it is widely used as an additive to piezoelectric biosensors to enhance sensitivity and mechanical performance. These biosensors, particularly piezo-resistive types, operate by converting external mechanical forces into measurable changes in electrical resistance. When strain is applied, the sensor’s conductance changes, and deformation of the sensor alters its geometry, further affecting resistance [2,23]. In designing piezoelectric biosensors, key parameters such as stretchability, sensitivity, dynamic range, limit of detection, accuracy, response time, stability, durability, fabrication cost, and simplicity should all be considered. Graphene’s outstanding mechanical strength, flexibility, and large surface area make it an ideal material for coating or integrating with conventional piezoelectric substrates. It has also contributed to significant advancements in micro- and nano-electro-mechanical sensing (MEMS and NEMS) technologies. In mass-sensitive biosensors, such as quartz crystal microbalance (QCM) devices, graphene functionalisation enhances biomolecule immobilisation, leading to improved analyte binding accuracy. For strain-sensitive applications, graphene’s ability to withstand large deformations without losing conductivity significantly improves the detection of mechanical changes triggered by biological interactions [42,43,89].

#### 4.2.1. Mass-Sensitive Piezoelectric Biosensors

**Figure 4:**
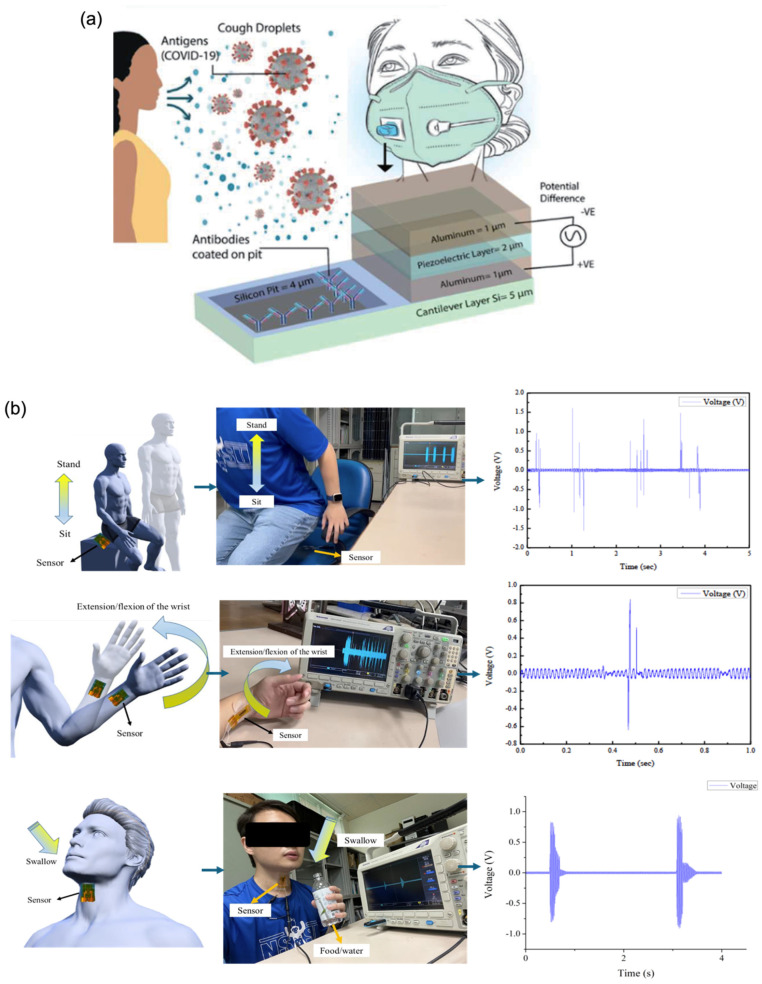
Wearable piezoelectric biosensors. (**a**) Three-dimensional schematic of a mass-sensitive bio-MEMS sensor with a piezoelectric layer (Reprinted with permission from Ref. [24]. Copyright 2021, © IEEE). (**b**) Potential wearable and biomedical applications of a graphene-doped piezoelectric silk sensor [58].

Mass-sensitive piezoelectric biosensors operate by detecting frequency shifts induced by the adsorption of biomolecules onto the sensor surface [90]. When analytes bind to the functionalised surface, the resultant increase in mass alters the resonant frequency of the underlying piezoelectric material, a change that can be quantitatively measured. This method enables highly sensitive detection of even small amounts of biological material. Graphene enhances the performance of these biosensors through its high surface area, mechanical flexibility, and strong affinity for biomolecule immobilisation. These properties contribute to increased interaction with target analytes and enable the detection of minute mass changes with high precision. A compelling example is presented by Abdullah et al. [24], who designed a simulated piezoelectric MEMS-based biosensor integrated into a wearable face mask for the detection of *SARS-CoV-2* virus droplets (Figure 4a). The system incorporated antibody-coated cantilever surfaces functionalised with graphene. Upon binding of viral particles, an electric potential was generated, indicating successful detection. Remarkably, the biosensor demonstrated the ability to detect individual virions with a radius of 0.05 μm and a mass as low as 1 femtogram (fg), underscoring its ultra-sensitive capabilities and potential for real-time, wearable viral diagnostics.

#### 4.2.2. Strain-Sensitive Piezoelectric Biosensors

Strain-sensitive piezoelectric biosensors detect mechanical deformations such as bending, stretching, or compression, induced by biological interactions. When a target analyte binds to the functionalised surface, it generates a mechanical strain that is transduced into an electrical signal via the piezoelectric effect. This mode of sensing is particularly advantageous for wearable and real-time monitoring applications. Graphene significantly enhances the performance of these biosensors due to its exceptional mechanical flexibility, high tensile strength, and outstanding electrical conductivity [43,91]. These properties allow for the detection of subtle deformations with high sensitivity and enable rapid and efficient signal transduction. To further support flexibility and integration into wearable devices, graphene can be deposited onto or blended with stretchable polymer substrates such as polydimethylsiloxane (PDMS) and thermoplastic polyurethane (TPU) [23]. A representative example is the development of a piezoelectric biosensor based on near-field electrospinning of a polyvinylidene fluoride (PVDF) matrix doped with graphene, designed to monitor body movements and swallowing activity (Figure 4b). While pure PVDF exhibits piezoelectricity, its output signals are typically weak and often require additional circuitry for amplification. However, doping PVDF with 5 wt% graphene resulted in a significant enhancement in performance, yielding a peak output voltage of 4.56 V, approximately 11.54 times higher than that of undoped PVDF. This underscores graphene’s potential in enabling highly responsive, flexible biosensors suitable for physiological monitoring and human–machine interfacing [58].

### 4.3. Electrochemical Biosensors

EC biosensors are highly regarded for their sensitivity, rapid response, and compatibility with miniaturised platforms, making them ideal for portable, low-power, and cost-effective analytical devices. These sensors function by converting biochemical interactions such as enzyme–substrate reactions or biomolecular binding events into measurable electrical signals, enabling the detection of a wide range of analytes [77,92,93,94]. EC biosensors are typically categorised into three main types based on their signal detection mechanisms:

(1) Impedimetric biosensors, which detect changes in electrical impedance (resistance and capacitance) at the electrode–electrolyte interface.

(2) Amperometric biosensors, which measure the current generated at a constant applied potential due to redox reactions.

(3) Voltammetric biosensors, which monitor current responses over a range of applied potentials to identify electroactive species [7,10].

Graphene-based electrodes significantly improve the performance of all three EC biosensor types. Due to graphene’s extraordinary electrical conductivity, large electroactive surface area, and chemical tunability, it facilitates more efficient electron transfer at the sensor interface, thereby enhancing sensitivity, stability, and detection limits. Moreover, graphene’s ability to be functionalised with specific receptors or nanostructures further broadens its application in detecting clinically and environmentally relevant targets with high specificity and low background noise [25].

#### 4.3.1. Impedance-Based Biosensors

Impedance-based biosensors, commonly implemented through EC Impedance Spectroscopy (EIS), evaluate the resistance of an EC system to an applied alternating current (AC). When biomolecular interactions occur at a graphene-modified electrode surface, they induce alterations in charge transfer resistance and double-layer capacitance, thereby modifying the impedance profile of the system [95]. These sensors are generally classified into two types:

(1) Conductive impedance biosensors, which detect changes in electrical resistance resulting from analyte-binding events at the electrode interface.

(2) Capacitive impedance biosensors, which monitor variations in dielectric properties caused by changes in the insulating layer such as its dielectric constant and thickness upon analyte binding [96].

In both cases, binding events are reflected as shifts in the impedance spectrum across a range of frequencies, enabling label-free, non-invasive, and highly sensitive detection of biomolecular interactions. Graphene offers several key advantages for impedance biosensing. Its exceptional electrical conductivity enhances charge transfer kinetics, while its large surface area and chemical functionality provide abundant sites for stable bioreceptor immobilisation. These characteristics contribute to improved signal-to-noise ratios and lower detection limits, which are particularly useful in complex biological environments. Importantly, impedance biosensors, especially those operating in non-faradaic electrochemical impedance spectroscopy (nf-EIS) mode, do not require redox-active species or external labels. nf-EIS measures changes in interfacial capacitance and resistance without electron transfer reactions, which helps reduce background noise, improves measurement reproducibility, and simplifies device fabrication [97,98,99,100].

Recent advances in impedance-based biosensors have demonstrated the feasibility of low-cost, portable, and highly sensitive platforms for real-time diagnostics. For example, a handheld, reconfigurable impedimetric readout system was developed for viral diagnostics, offering an affordable solution with impressive performance characteristics [101]. The system featured a dynamic input range of 200 Ω to 1 MΩ, with a magnitude resolution of 1% and phase resolution of 6.5° across a 0° to 180° range. It demonstrated sensitivity for detecting viral nucleocapsid (N) protein concentrations up to 10,000 pg/mL, with a lower resolution limit of 56 fg/mL, underscoring its utility for early-stage viral detection. In another innovative application, a graphene electronic tattoo (GET)-based biosensor was employed in a proof-of-concept device for continuous, non-invasive blood pressure monitoring. The device deployed three pairs of GET electrodes over the radial and ulnar arteries on the wrist. Alternating current (0.2 to 1 mA_AC) at 10 kHz was injected through the outer terminals, while the inner terminals recorded the resulting biopotential variations. The signal was subsequently bandpass-filtered and demodulated using a low-noise, multichannel sensing platform. This setup enabled bioimpedance detection with a resolution down to 1 mΩ and achieved blood pressure measurements with accuracies of 0.2 ± 4.5 mmHg (diastolic) and 0.2 ± 5.8 mmHg (systolic), comparable to Grade A medical devices. These examples highlight the versatility and scalability of graphene-enabled impedance biosensors, capable of supporting both clinical diagnostics and wearable health monitoring applications. The integration of graphene enhances signal fidelity, device flexibility, and long-term biocompatibility, key factors for next-generation, real-world biosensing technologies [102].

#### 4.3.2. Amperometric Biosensors

Amperometric biosensors operate by measuring the electrical current produced during redox reactions of target analytes at a constant applied potential. The magnitude of the resulting current is directly proportional to the analyte concentration, allowing for precise quantitative analysis. However, their high sensitivity also renders these sensors more vulnerable to electrical noise and environmental interference [28,103].

Graphene significantly enhances the performance of amperometric biosensors due to its exceptional electron mobility, which facilitates rapid charge transfer at the electrode interface. This results in improved sensitivity and reduced detection limits [104]. In contrast to impedance-based sensors, which are typically label-free, amperometric biosensors require electroactive analytes capable of participating in oxidation or reduction reactions, which confer high specificity but limit the sensors to redox-active targets [96]. In a notable study, Liu et al. [105] developed a laser-induced graphene (LIG) electrode by laser-engraving polyimide, followed by crosslinking with a bovine serum albumin–glucose oxidase (BSA–GOx) enzyme complex. The resulting glucose biosensor exhibited high sensitivity, a broad linear detection range, and a low limit of detection. Importantly, the sensor retained conductivity under bending angles up to 60°, demonstrating its potential for integration into flexible and wearable biosensing platforms. Another example by Zhu et al. [106] highlighted the strong selectivity of a graphene-based amperometric sensor for detecting a variety of non-enzymatic analytes, including ascorbic acid, dopamine, uric acid, sodium chloride, citric acid, and glucose. The sensor achieved a limit of detection of 0.032 ± 0.005 μM at a low operating potential of –0.55 V, with a signal-to-noise ratio of 3. The low working potential reduces power consumption and simplifies circuit design, which is advantageous for portable applications. Additionally, graphene exhibits intrinsic electrocatalytic activity toward redox-active molecules such as hydrogen peroxide, enabling non-enzymatic detection strategies. This eliminates the need for enzyme immobilisation, simplifying fabrication and improving long-term sensor stability, especially under variable environmental conditions.

#### 4.3.3. Voltammetric Biosensors

Voltammetric biosensors measure variations in electrical current in response to a time-dependent applied potential, offering powerful insights into redox behaviour and electrochemical kinetics. Widely employed techniques include cyclic voltammetry (CV), square wave voltammetry (SWV), and differential pulse voltammetry (DPV), each optimised for specific analytical contexts [29,107]. CV involves a linear potential sweep in both forward and reverse directions, producing a characteristic current–potential (I–V) curve with distinct oxidation and reduction peaks that reveal redox activity and reversibility [27,108].

SWV overlays square wave potential pulses onto a staircase waveform, measuring the net current between forward and reverse pulses to enhance signal discrimination [70]. DPV, in contrast, applies small amplitude potential pulses on a linearly increasing baseline, capturing current differentials before and after each pulse to maximise sensitivity [109]. These methods offer complementary advantages: CV excels in probing redox mechanisms and electron transfer kinetics, DPV is preferred for trace-level detection due to its high sensitivity and low background current, and SWV enables rapid, sensitive screening with minimal signal overlap. Upon interaction with the target biomolecule, the sensor exhibits quantifiable changes in peak current intensity and potential shifts, which directly correlate with analyte concentration and binding affinity [68]. The integration of graphene into voltammetric platforms significantly elevates performance, thanks to its exceptional conductivity and large electroactive surface area. This leads to sharper peak resolution, enhanced signal-to-noise ratios, and overall improved detection limits. Compared to amperometric sensors, voltammetric techniques deliver richer electrochemical profiles and mechanistic insights, albeit with more complex data interpretation requirements [10]. Nonetheless, their versatility and analytical depth make them indispensable tools in modern biosensing applications.

#### 4.3.4. Advances in Portability

The rapid advancement of miniaturised electronics has paved the way for portable voltammetric biosensors, enabling real-time, on-site diagnostics that rival traditional laboratory-based systems. These compact platforms integrate microfabricated components, wireless data acquisition, and user-friendly interfaces, making them ideal for field deployment and POC applications. A notable example is the work by Challhua et al. [110], who developed a portable biosensor for the detection of rabies virus (RABV) in bat nasopharyngeal swab samples. The sensor employed a standard three-electrode configuration, utilising rGO as the working electrode, while gold electrodes served as both the reference and counter. Cyclic voltammetry was performed using a handheld potentiostat, with immobilised RABV cDNA on the rGO surface to ensure target specificity. The biosensor achieved a limit of detection (LOD) of 0.104 ng/μL, a sensitivity of 0.321 μA (ng/μL)^−1^, and a linear detection range of 0.145–25.39 ng/μL. Remarkably, its analytical performance was comparable to standard RT-PCR assays, underscoring the viability of voltammetric platforms for rapid, low-cost, and decentralised viral diagnostics. Such developments mark a significant leap toward democratising advanced diagnostics, particularly in low-resource or remote settings where conventional lab infrastructure is inaccessible.

#### 4.3.5. Modular Architecture of Electrochemical Biosensors

**Figure 5:**
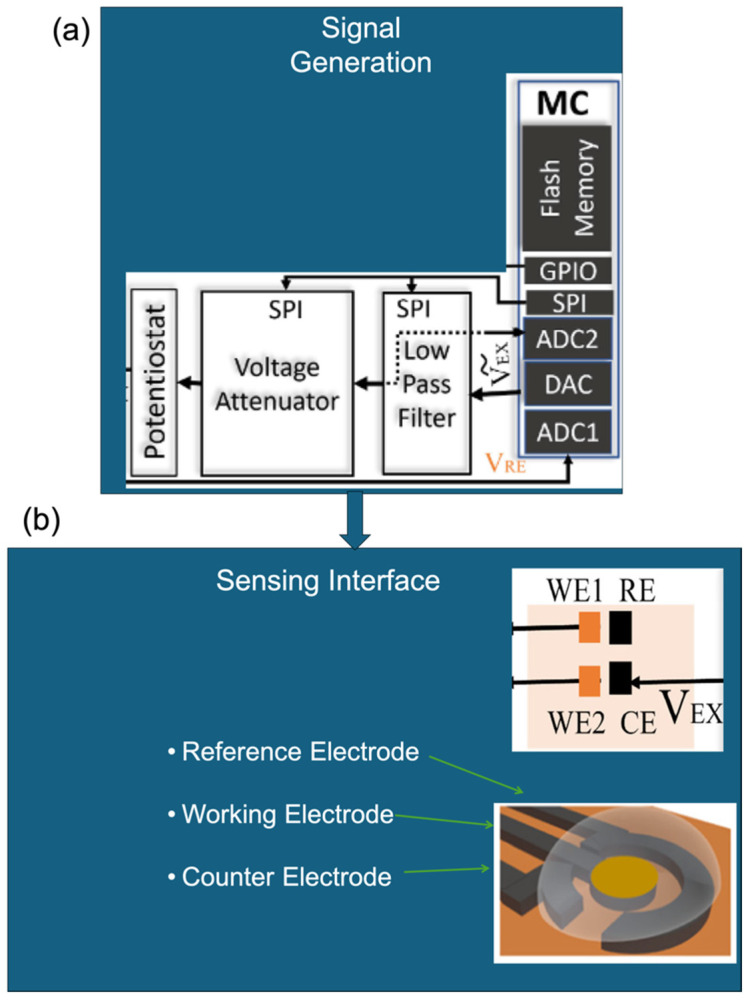

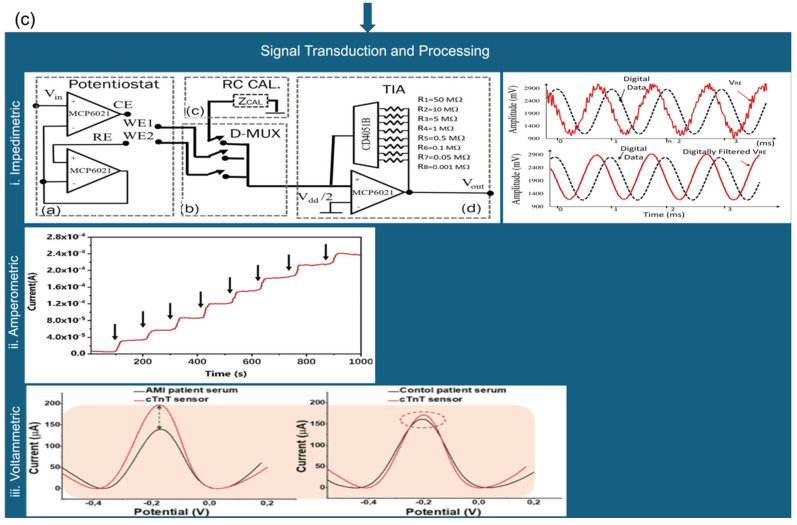
Modular block diagram of EC biosensors showing (**a**) signal generation (adapted with permission from Ref. [101]. Copyright 2023, © IEEE), (**b**) sensing interface (three-electrode setup) (Adapted with permission from Ref. [101]. Copyright 2023, © IEEE) and [109], and (**c**) signal transduction. Characteristic signal profiles for (ci) impedimetric (Adapted with permission from Ref. [101]. Copyright 2023, © IEEE), (cii) amperometric (Adapted with permission from Ref. [105]. Copyright 2023, Elsevier), and (ciii) voltammetric methods are shown for comparison [109].

EC biosensors, whether impedimetric, amperometric, or voltammetric, commonly follow a modular design architecture, facilitating their adaptability and integration across diverse applications. Figure 5 provides an illustration of this modularity.

Figure 5a depicts the signal generation unit, typically comprising a potentiostat or microcontroller, which applies and controls the necessary EC stimuli. Figure 5b shows the widely adopted three-electrode configuration consisting of a working, reference, and counter electrode that forms the core of most EC biosensing platforms. Figure 5c outlines the distinctions in signal transduction and processing for each sensing modality:

(i) Impedimetric sensors monitor changes in impedance over time (Figure 5c(i)).

(ii) Amperometric sensors measure steady-state current at a fixed potential (Figure 5c(ii)).

(iii) Voltammetric sensors capture current responses during dynamic potential sweeps (Figure 5c(iii)).

While the hardware layers such as the electrode arrangement and control circuitry are largely standardised (Figure 5a,b), the signal processing pathways are modality-specific. Impedance spectra, fixed-potential current values, or current–voltage profiles each offer distinct analytical advantages depending on the target analyte and context. This modular design underpins the versatility and scalability of EC biosensors, enabling multi-modal sensing within a unified platform. It also supports seamless integration into portable, wearable, or networked diagnostic systems, thereby broadening their utility across biomedical, environmental, and industrial domains.

### 4.4. Transistor Biosensors

GFETs represent a significant advancement in biosensing, leveraging graphene’s extraordinary electrical, mechanical, and chemical properties. These include high sensitivity, excellent selectivity, low detection limits, and ultra-low power consumption, all of which are critical for next-generation diagnostic platforms [29,111].

**Figure 6:**
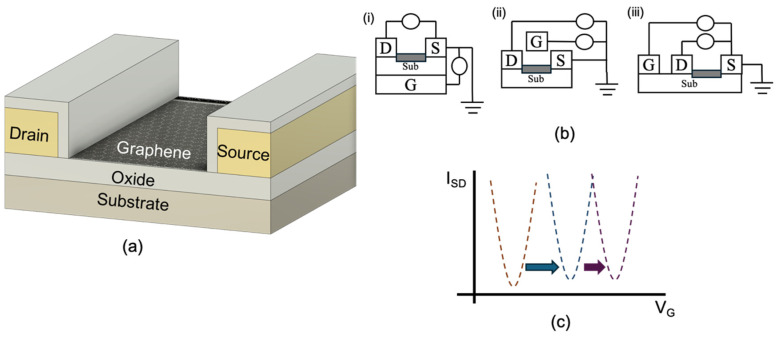
Graphene transistors’ (**a**) cross-section layout; (**b**) gating topologies: (i) back-gating, (ii) top-gating, and (iii) coplanar; and (**c**) sample Dirac point shifts (designed by the authors).

A standard GFET structure comprises a graphene channel positioned between source and drain electrodes, with a gate electrode used to modulate the carrier concentration within the channel. This gate modulation enables label-free, real-time monitoring of biochemical interactions, making GFETs highly attractive for biosensing applications. Figure 6a illustrates a cross-section of a commercially available GFET. In this configuration, chemical vapour deposition (CVD) graphene is transferred onto a silicon oxide (SiO_2_) substrate. The source and drain electrodes are fabricated using gold (Au) and subsequently encapsulated with aluminium oxide (Al_2_O_3_). This encapsulation serves to electrically isolate the electrodes from the sensing environment, ensuring that only the graphene channel participates in electrochemical interactions [112]. The performance of GFET biosensors is strongly influenced by the choice of substrate and dielectric layer, which affect critical factors such as charge trapping, carrier mobility, and mechanical stability [31,32]. Optimising these materials and interface conditions is essential for achieving reproducible and high-performance sensing in practical applications.

The positioning of the gate electrode in GFETs is a critical design parameter that directly affects device sensitivity, fabrication complexity, and applicability. Figure 6b illustrates the primary gate configurations used in GFET architectures, each tailored to specific operational requirements. In the back-gated GFETs configuration (Figure 6b(i)), the gate electrode is located beneath the substrate. While this design offers simplified fabrication, its sensitivity is typically lower due to the greater distance between the gate and the graphene channel, which weakens electric field coupling. The top-gated GFET (Figure 6b(ii)) improves electric field control by placing the gate electrode directly above the graphene channel, thereby enhancing modulation of carrier density. However, this configuration may limit physical access to the graphene surface, which can be a disadvantage in biosensing applications that require surface functionalisation or direct analyte interaction [113,114].

The coplanar gate configuration (Figure 6b(iii)), in which the gate electrode is positioned laterally adjacent to the graphene channel, strikes a balance between strong electric field control and unobstructed access to the sensing surface. This architecture is especially useful when multiple GFETs are controlled by a single gate voltage source, simplifying circuit integration [8]. In addition to solid-state configurations, liquid-gated or electrolyte-gated GFETs are widely used in biosensing. In these systems, an electrolyte solution serves as the gate dielectric, providing direct ionic coupling between the gate and the graphene surface. This enhances sensitivity and allows for real-time detection of biochemical interactions in aqueous environments [59,115]. Despite differences in gate orientation, the underlying working principle of all GFET configurations remains consistent: modulation of the graphene channel’s conductivity via gate-induced carrier density changes [113,116]. Given their miniaturised footprint, most GFET-based biosensors are integrated with microfluidic systems to precisely control the delivery and removal of liquid samples. PDMS is commonly used to fabricate microfluidic channels due to its biocompatibility, optical transparency, and ease of moulding. Microfluidic integration not only improves sample handling and reduces reagent consumption but also enables rapid, multiplexed detection, making GFET platforms particularly attractive for POC diagnostics. Nevertheless, challenges such as evaporation, adsorption of biomolecules to channel walls, and fabrication complexity can influence performance and reproducibility. Despite these limitations, microfluidics remains a key enabler in translating GFET-based biosensors into practical, real-world diagnostic solutions [80,95,117].

#### Dirac Point-Based Detection Mechanism in GFETs

The core sensing principle of GFETs lies in the shift in the Dirac point, the gate voltage at which graphene exhibits minimum conductivity, corresponding to the charge neutrality point (CNP) where electron and hole concentrations are equal. This unique behaviour stems from graphene’s sp^2^-hybridised lattice structure, which produces a zero-bandgap electronic profile, with conduction and valence bands intersecting at the so-called Dirac cones. The apex of these cones marks the CNP, also commonly referred to as the Dirac point [118].

While pristine graphene ideally displays the Dirac point at zero gate bias, real-world devices exhibit shifts in the CNP due to external perturbations such as chemical doping, electrostatic gating, and interfacial interactions [48,54]. In the biosensing context, binding of biomolecules to the graphene surface introduces localised charge transfer: electron-donating or electron-withdrawing analytes modulate the carrier density, thereby inducing a shift in the Dirac voltage. This shift directly reflects changes in the electronic environment of the graphene channel and thus serves as a highly sensitive and label-free indicator of molecular recognition events. Other factors including substrate-induced charge traps, surface adsorbates, and intentional doping strategies can also influence the Dirac point by disrupting the delicate balance of electron–hole symmetry [114,119]. These effects must be accounted for during device design and calibration. As illustrated in Figure 6c analyte binding causes a lateral shift in the Dirac curve, observable in the transfer characteristics (drain current vs. gate voltage). By comparing these curves before and after sample exposure, the presence and concentration of target biomolecules can be quantitatively determined.

The reliability and sensitivity of Dirac point-based detection depend not only on accurately capturing these voltage shifts but also on optimising the biofunctionalisation of the graphene surface. Stable and specific immobilisation of bioreceptors, combined with strategies to minimise environmental noise and signal drift, are essential to maximise performance. In summary, the Dirac point shift mechanism provides a direct, real-time, and ultra-sensitive approach for GFET-based biosensing, capitalising on graphene’s distinctive electronic properties to enable next-generation analytical platforms [35,120].

## 5. Multiplex Detection

Multiplexed graphene biosensing has emerged as a transformative approach for the simultaneous detection of multiple analytes, leveraging graphene’s exceptional electrical conductivity, high surface-to-volume ratio, and tunable surface chemical. Through advances in electronic microfabrication and surface functionalisation, graphene-based platforms can be miniaturised to enable multi-target detection within a compact footprint, significantly enhancing throughput, sensitivity, and specificity in biosensing applications. Two primary strategies dominate multiplex graphene biosensor design: array-based configurations, and multireceptor functionalisation on a single graphene surface, each with distinct advantages and limitations [121,122,123].

In array-based platforms, multiple discrete graphene biosensors are individually functionalised with different bioreceptors, each tailored to a specific analyte or pathogen. These sensors are electrically isolated but controlled by a shared measurement system, often employing a multiplexer to sequentially read from each sensing unit. This configuration simplifies hardware requirements, needing only a single control circuit, and allows for modular scaling of sensor arrays. However, sequential readout can reduce measurement speed, particularly when analysing large numbers of targets, and careful calibration is required to maintain uniform sensitivity across all channels. Alternatively, multi-functionalised single-surface systems involve the immobilisation of multiple bioreceptor elements on distinct regions of a single graphene sheet. This enables truly parallel detection, as individual analytes generate spatially or spectrally distinguishable signals that can be independently monitored. In electrical platforms, responses may be differentiated using predefined voltage thresholds, frequency-dependent impedance, or field-effect transistor characteristics, whereas in optical platforms, spectral separation or intensity differences in fluorophores or Raman-active labels are used. While less common in electrical biosensors, this approach is widely applied in optical detection, such as fluorescence-based or graphene-enhanced Raman spectroscopy (GERS) systems [124,125,126,127,128,129].

For example, GERS exploits graphene’s signal-amplifying properties to enable label-free identification of multiple analytes based on their unique Raman spectral fingerprints, while fluorescence-based platforms can assign distinct fluorophores to each target analyte, allowing simultaneous detection from mixed sample [127]. These examples illustrate how single-surface multiplexing can achieve high-throughput detection without significantly increasing device footprint. Overall, multiplexed graphene biosensors offer a versatile and efficient platform for high-throughput, real-time, and miniaturised diagnostics. Their capacity to interrogate complex biological or environmental samples with high fidelity, combined with the flexibility to tailor array size and surface functionalisation, makes them particularly attractive for clinical diagnostics, environmental surveillance, food safety, and biodefense applications.

### 5.1. Single Graphene–Single Bioreceptor Architecture

The most widely adopted approach for multiplexed detection involves fabricating individual graphene biosensors, each functionalised with a single bioreceptor, and arranging them into an array configuration. This modular structure enables the simultaneous detection of multiple analytes on a single device by assigning each sensor to a specific target [121]. Alternatively, a device may be dedicated to a single analyte while incorporating multiple identical biosensors to enable statistical validation and redundancy, improving reliability and robustness in critical applications. These graphene sensor arrays are typically interfaced with a microcontroller unit (MCU) to facilitate rapid, parallel measurements. Depending on application requirements, graphene electrodes may be read directly by the MCU or routed through amplification and signal isolation stages to enhance measurement accuracy. In the case of GFET-based biosensors, the intrinsic transistor characteristics necessitate amplifiers for signal conditioning, followed by multiplexers to sequentially access signals from each GFET channel. The analogue outputs are then converted to digital form using an analogue-to-digital converter (ADC) before being processed by the MCU [8,13,125].

The selection of components including amplifiers, multiplexers, and ADCs is influenced by various design constraints such as the following:

(a) Commercial availability and cost.

(b) Component count and printed circuit board (PCB) footprint.

(c) Signal integrity across transducer-to-ADC distance.

(d) Power consumption and voltage compatibility.

(e) MCU pin availability and computational overhead.

(f) Environmental or testing conditions.

**Figure 7:**
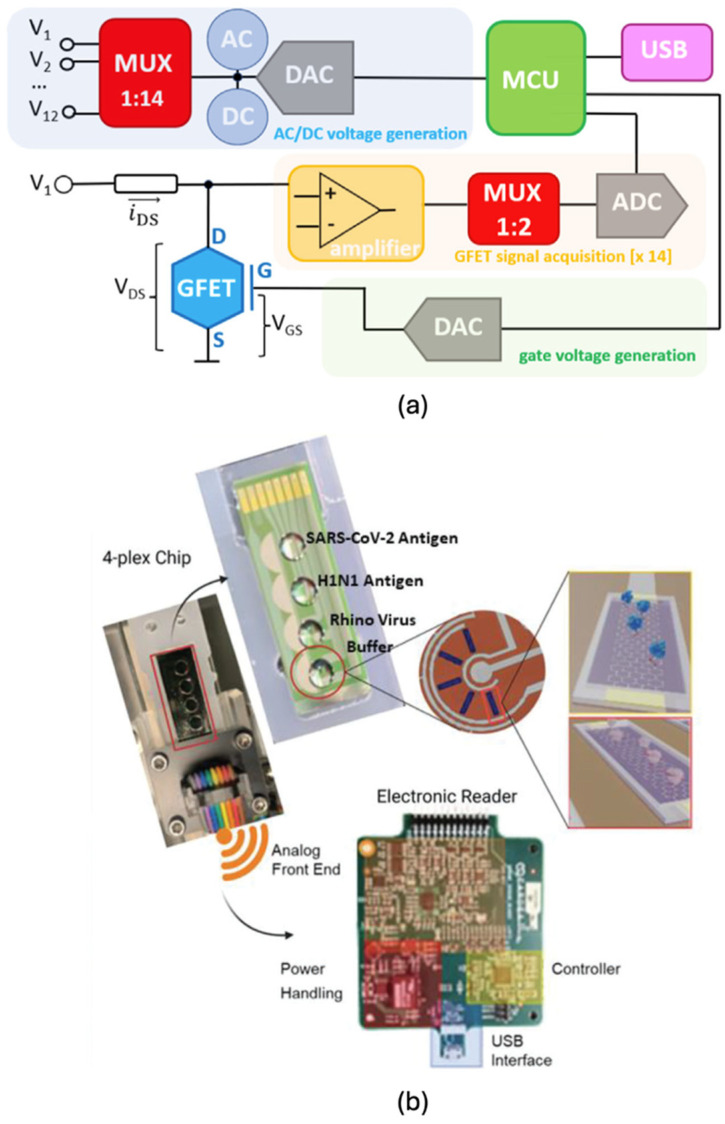
Multiplexed array graphene sensing (**a**) sample circuit (Reprinted with permission from Ref. [130]. Copyright 2022, Elsevier) and (**b**) sample diagnostic device components [131].

An illustrative implementation of this architecture was presented by Gil et al. [130], who developed a liquid-gated GFET array for multi-analyte biosensing. Their platform integrated multiple GFETs on a single silicon substrate, designed for low-cost and scalable production. The system architecture, shown in Figure 7a, features one multiplexer for delivering gate or drain voltages to individual GFETs and a second multiplexer for collecting amplified output signals. These signals are routed through an ADC and subsequently processed by a microcontroller. The study also demonstrated robust mathematical modelling and evaluated the system’s performance across various solution conductivities, highlighting its applicability in diverse biosensing scenarios. This architecture exemplifies a scalable and versatile framework for real-time, high-precision detection of multiple targets using graphene-based platforms.

### 5.2. Portable Multiplex Devices for Disease Detection

The integration of multiplex graphene biosensors into portable platforms has opened new frontiers for on-site, real-time diagnostics, particularly in the detection of infectious diseases. These compact, multi-analyte systems combine the sensitivity of graphene-based transducers with advanced biochemical and computational tools for robust performance in diverse environments.

A notable example is the multiomic transistor platform developed by Ban et al. [131], designed for the simultaneous detection of *SARS-CoV-2* antigens and viral RNA variants. The system employed protein-catalysed capture (PCC) bioreceptors for antigen detection and a chimeric RNA-DNA probe coupled with LwaCas13a (Leptotrichia wadei CRISPR-associated protein 13a) for RNA sensing on a GFET array. The biosensor achieved limits of detection (LOD) of 10^3^ PFU/mL in buffer and 10^4^ PFU/mL in 10% saliva. To enhance the accuracy and robustness of the platform, principal component analysis (PCA) was applied as a machine learning technique to distinguish signal patterns and improve classification reliability. The biosensor chip integrated four distinct wells (Figure 7b), configured to detect *SARS-CoV-2* antigen, *H1N1* antigen, rhinovirus, and a buffer control. The system also incorporated essential components of a portable diagnostic tool: an electronic reader for GFET outputs, an analogue front-end, power management module, microcontroller, and USB communication interface.

In a similar development, Geiwitz et al. [125] demonstrated a graphene electronic multiplexed sensor (GEMS) array capable of being mass-fabricated from a 4 inch wafer, yielding 44 individual chips, each with 20 GFETs grouped into four analyte-specific sets. This platform was used to monitor viral pathogens in wastewater, including *SARS-CoV-2*, respiratory syncytial virus (RSV), influenza A, and a population normalisation marker (caffeine). Impressively, the reported limits of detection were 453 ag/mL (RSV), 408 ag/mL (influenza A), 55 ag/mL (*SARS-CoV-2*), and 26 fg/mL (caffeine), highlighting the system’s potential for public health surveillance in decentralised settings.

A high-sensitivity, disease-specific approach was also demonstrated by Beduk et al. [109], who fabricated a laser-scribed graphene (LSG) biosensor modified with nanostructured gold for the simultaneous detection of acute myocardial infarction (AMI) biomarkers. The device incorporated three individual LSG electrodes, each functionalised to detect cardiac troponin T (cTnT), cardiac troponin I (cTnI), and C-reactive protein (CRP). The biosensor achieved detection limits of 1.65 ng/mL (cTnT), 2.58 ng/mL (cTnI), and 1.84 ng/mL (CRP), demonstrating the efficacy of parallel, single-electrode readout in enhancing multiplex performance. Collectively, these examples underscore the growing sophistication and versatility of portable graphene-based multiplex sensors in addressing urgent biomedical and environmental challenges. Their high sensitivity, compact integration, and scalability position them as promising candidates for next-generation diagnostic technologies.

### 5.3. Single Graphene–Multiple Bioreceptors

A more sophisticated strategy for multiplexed biosensing involves functionalising a single graphene layer or channel with multiple bioreceptors, each selective for a different analyte. This approach significantly reduces device complexity and footprint while enabling simultaneous, multi-target detection on a single sensing platform. However, it requires precise control of spatial functionalisation, as well as in-depth understanding of the chemical and biological interactions between analytes, receptors, and the graphene interface [53,132].

One representative implementation is a GO- based sensing platform designed for the multiplexed detection of protein kinases using dye-labelled peptide substrates. The detection process involves kinase-mediated phosphorylation of the peptide substrate in the presence of biotinylated adenosine triphosphate (ATP). The phosphorylated peptides, now carrying both biotin and a fluorophore, are assembled onto streptavidin-coated GO via strong biotin–streptavidin affinity, resulting in fluorescence quenching. By utilising multicolour fluorescent peptide probes, this system enables homogeneous multiplexed detection of protein kinases in solution. Huang et al. reported high selectivity and low detection limits for protein kinase A (0.005 U/mL), Abl (0.02 U/mL), and Src (0.05 U/mL). The authors also demonstrated that the platform is easily extensible to other kinases by substituting the appropriate peptide substrates [133].

Another elegant optical method leverages aptamer-functionalised GO for multiplex detection of antibiotics, using a fluorescence resonance energy transfer (FRET) strategy coupled with DNase I-assisted cyclic enzymatic signal amplification (CESA) [39]. In this platform, aptamers specific for sulfadimethoxine, kanamycin, and ampicillin were conjugated with distinct fluorophores—Cyanine 3 (Cy3), 6-carboxyfluorescein (FAM), and Cyanine 5 (Cy5), respectively. Upon specific binding to the target analytes, fluorescence signals were restored following desorption from GO and DNase I-driven cleavage. The reported limits of detection were 1.997 ng/mL for sulfadimethoxine, 2.664 ng/mL for kanamycin, and 2.337 ng/mL for ampicillin. The platform demonstrated successful simultaneous detection of all three antibiotics in real-world milk samples, underscoring its potential for practical food safety monitoring.

These examples illustrate the immense potential of single-surface, multireceptor graphene platforms for developing highly integrated, compact, and sensitive multiplexed biosensors. Such systems are particularly attractive for POC diagnostics, environmental surveillance, and food quality assurance, where simultaneous detection of multiple targets is essential.

### 5.4. Linker-Enabled Multiplex Detection on a Single Medium

An alternative strategy for multiplexed detection on a single graphene-based medium involves the design and selection of molecular linkers to enable the co-immobilisation of multiple bioreceptors. These functional linkers selectively bind to graphene edges or basal planes, altering surface chemistry and enabling site-specific attachment of diverse biomolecular probes. This approach allows a single graphene interface to support simultaneous detection of multiple analytes, while preserving signal fidelity and minimising cross-interference [68,72,134].

**Figure 8:**
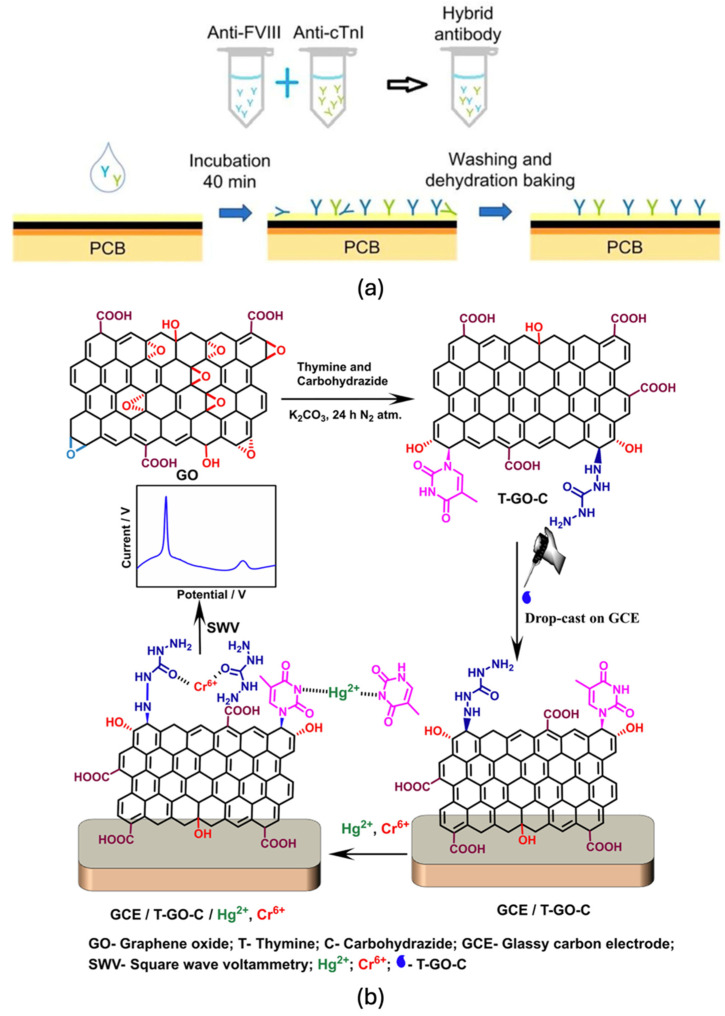
Biofunctionalisation process for (**a**) a direct [53] and (**b**) a linker-based multi-bioreceptor immobilisation approach (Reprinted with permission from Ref. [68]. Copyright 2022, Elsevier).

A compelling example of this approach was reported by Chang, Siao, and Lin [53], who developed a GO-based biosensor functionalised with silk fibroin as a linker for the simultaneous detection of coagulation factor VIII (FVIII) and cardiac troponin I (cTnI). These biomarkers are critical in distinguishing between pulmonary embolism (PE) and myocardial infarction (MI). As shown in Figure 8a, the biosensor was co-functionalised with antibodies against both biomarkers. Linear sweep voltammetry (LSV) was employed for detection, and the curvature coefficients derived from the LSV curves were used to classify plasma samples. The study found non-overlapping coefficient values for FVIII and cTnI, confirming that the biosensor could accurately detect and differentiate between PE and MI. Notably, no signal superposition was observed when both biomarkers were present, indicating effective decoupling of their electrochemical signatures.

A related multiplex sensing approach was demonstrated by Jayaraman et al. [68], who developed a dual-analyte electrochemical sensor capable of detecting Hg(II) and Cr(VI) simultaneously. The GO surface was covalently functionalised with thymine and carbohydrazide via epoxide ring cleavage. This chemical modification strategy enabled specific recognition of the metal ions based on well-characterised binding interactions: thymine formed strong coordination complexes with Hg(II) through thymine–thymine base pairing, while carbohydrazide selectively bound to Cr(VI). As shown in Figure 8b, the sensor exhibited distinct, linear responses for each analyte above 5 ppb, with limits of detection of approximately 1 ppb for Hg(II) and 20 ppb for Cr(VI). These results validated the linker-enhanced functionalisation strategy as a viable means for dual-metal ion detection with good selectivity and sensitivity. Overall, linker-enabled surface modification provides a versatile, chemistry-driven approach to multiplex biosensing on graphene platforms. By tailoring surface affinity and selectivity, researchers can design customised detection interfaces for complex biological or environmental samples while maintaining signal resolution and minimising cross-reactivity. This approach holds significant promise for multi-marker diagnostics, pollutant screening, and resource-efficient analytical systems.

### 5.5. Evolving Strategies in Multiplex Detection

An innovative approach for multigas identification using a single carbon-based transistor was proposed by Shi et al. (2024) [135], in which CNTs were deposited onto a silicon substrate to fabricate a CNT field-effect transistor (CNT-FET). The device was functionalised with palladium (Pd) nanoparticles to enhance gas sensitivity. By monitoring eight electrical parameters, including threshold voltage (V_th_), transconductance (g_m_), and drain current (I_ds_), across multiple voltage levels and applying PCA, the system successfully distinguished between (NO_2_), (NH_3_), (H_2_), (H_2_S), CO, and (SO_2_). Detection was achieved at concentrations as low as 1 ppm for most gases and 200 ppm for NH_3_, with good reproducibility. Although the study did not report precise sensitivity or accuracy metrics, the use of PCA and electrical fingerprinting demonstrates the potential of multivariate analysis to enhance the selectivity of multiplexed FET-based gas sensors. While this work specifically employed CNT-FETs, the same methodology could be readily extended to graphene-based FETs, which offer superior carrier mobility and larger surface area for functionalisation, thereby holding promise for next-generation multiplexed gas detection. This highlights a future research opportunity to adapt PCA-based electrical fingerprinting strategies to graphene devices for improved gas-sensing performance. One noted limitation, however, is the wide gate voltage range (−60 V to +100 V), which may hinder portability and increase system complexity [135].

Notably, earlier multiplex detection strategies largely relied on fluorescence and spectral techniques, utilising spatial separation and spectral discrimination to identify multiple analytes. However, recent trends increasingly exploit the structural and electronic properties of graphene, enabling the integration of multiple bioreceptors on a single graphene sheet or channel. This shift reflects the growing sophistication in graphene device fabrication and surface engineering.

Among the various strategies, spatially resolved (array-based) multiplexing remains the most widely adopted due to its high selectivity, sensitivity, and reduced cross-reactivity [136,137,138]. Yet, advances in micro- and nanoscale patterning have made single-channel multiplexing a promising alternative, offering benefits in miniaturisation, integration, and cost-efficiency.

Across a wide range of transduction platforms including optical, piezoelectric, electrochemical, and field-effect transistor (FET)-based systems, graphene integration has consistently enhanced sensitivity, transduction efficiency, and device miniaturisation. The maturation of multiplex detection techniques, particularly in combination with graphene’s unique properties, now enables the simultaneous detection of multiple analytes using both spatially separated and multi-functionalised sensor surfaces.

A comprehensive overview of these multiplex detection mechanisms, bioreceptor integration strategies, and performance metrics across various graphene-based biosensing platforms is summarised in Table 2 and Table 3.

## 6. Conclusions and Future Perspectives

This review has highlighted the exceptional versatility of graphene-based biosensors, driven by graphene’s unique combination of structural, electrical, mechanical, and chemical properties. These attributes enable the development of highly sensitive, selective, and miniaturisable sensing platforms suitable for a wide range of biomedical and environmental applications. Central to the performance of graphene biosensors is effective biofunctionalisation, encompassing critical steps such as surface preparation, functional group attachment, bioreceptor immobilisation, and blocking of non-specific sites. Optimising these stages ensures signal stability, specificity, and reproducibility. Multiple detection modalities have been explored, each leveraging graphene’s properties in distinct ways:

In optical- and surface-based techniques, such as SPR, PL, and SERS, graphene serves as both a signal enhancer and quenching layer, benefiting from its large surface area and unique electronic interactions.

In piezoelectric sensors, graphene’s mechanical strength and high surface-to-volume ratio improve the sensitivity to minute mass or strain changes.

EC platforms including impedimetric, amperometric, and voltammetric biosensors benefit from graphene’s superior conductivity and electrocatalytic properties, which contribute to lower detection limits and faster response times.

GFETs have emerged as particularly promising tools for label-free, real-time detection. The review explored the impact of gate configurations with back-gated GFETs offering fabrication simplicity, and top- and coplanar-gated designs enhancing sensitivity at the expense of complexity. Liquid-gated GFETs, meanwhile, provide a balanced trade-off between sensitivity and sample accessibility, albeit requiring precise fabrication and system standardisation.

Moreover, the implementation of multiplex detection strategies has significantly expanded the diagnostic capabilities of graphene biosensors. This paper examined both of the following:

Array-based architectures, where spatially separated sensors enable parallel analyte detection.

Single-surface multiplexing, which uses combinatorial functionalisation to achieve multi-target recognition on a single graphene interface.

Critical enabling components such as custom microcontroller units (MCUs), signal amplification circuits, and ADCs were discussed in the context of system integration, highlighting the importance of circuit design and readout strategies in practical applications.

While graphene biosensors show strong promise, several challenges still hinder their large-scale commercialisation. Achieving reproducible large-scale synthesis, ensuring long-term operational stability, and integrating devices reliability into portable platforms remain major bottlenecks.

Standardised fabrication and characterisation protocols are needed to improve consistency across devices, while robust biofunctionalisation strategies must be developed to guarantee selectivity and biocompatibility over time. Moreover, scaling up manufacturing in a cost-effective manner will require close coordination between materials science, instrumentation, and biomedical engineering.

Addressing these challenges is crucial for transitioning graphene biosensors from laboratory demonstrations to viable commercial products.

In conclusion, graphene biosensors represent a rapidly evolving and multidisciplinary field, with strong potential for next-generation diagnostic tools. Future research directions should focus on the following:

Scalable and reproducible fabrication methods.

Standardised and stable biofunctionalisation protocols.

Low-power, portable electronics for real-time sensing.

Improved data analysis through machine learning and advanced signal processing.

Strategies for overcoming commercialisation barriers, including long-term stability, reproducibility, and manufacturing scalability.

Continued advancements along these lines will be essential to transitioning graphene biosensors from laboratory prototypes to real-world, POC platforms, where they can make a tangible impact on global health, environmental sustainability, and industrial monitoring.

## Figures and Tables

**Table 1:** Strategies for Graphene Biofunctionalisation.

Strategy	Sub-Strategy	Application	Bioreceptor	Immobilisation	Blocking	Ref.
**Covalent**	Amide coupling	H5N1, H7N9, and H9N2	antibodies	Crosslinker (EDC/NHS) for bonding of COOH-CNTs	BSA	[58]
		*E. coli*	*E. coli* antibody	NH_2_-GO and EDC/NHS	Tween 20	[64]
		*Salmonella Typhimurium*	polyclonal antibody	EDC/NHS	Superblock	[65]
	Amide coupling and Click Chemistry	CTCs and CTM	EpCAM and CSV Monoclonal Antibody	EDC/NHS and DBCO-PEG4-amine	N/A	[66]
		NPC CTC	anti-EpCAM	NH_2_-functionalised bilayer graphene and EDC/NHS	N/A	[67]
	Epoxide Ring-Opening	Hg(II) and Cr(VI)	Thymine and Carbohydrazide	T-GO-C	N/A	[68]
	Silanisation and Amide coupling	SARS-CoV-2 Antigen	*SARS-CoV-2* mAb	APTES and EDC/NHS	N/A	[69]
	Thiol–Gold Chemistry	miRNA-21, -155, and -210	AQ, MB, and PDA	dye/AuNPs/GO/GQD	MCH	[70]
	Thiol–Gold Chemistry, SAM, then Amide Coupling	DENV 2, DENV 1, and ZIKV E-proteins	IgMs	Au/DSU/amine-functionalised rGO–PAMAM/IgM and EDC/NHS	N/A	[71]
**Hybrid**	Non-covalent (π–π Stacking) and Covalent	SARS-CoV-2 spike protein and human ferritin	SARS-CoV-2-mAb and FTH1-mAb	Linker (PBASE-PEI) and Binding (DVS)	PEG and ETA	[54]
**Non-covalent**	Adsorption	PE (FVIII) and MI (cTnI)	mix anti-FVIII and anti-cTnI antibodies	Direct	Tween-20 buffer	[53]
	Electrostatic Adsorption	AIV biomarkers (H1N1, H7N9, and H5N1)	B–H2, B–H4, and B–H6	CHA-amplified Lum/PEI/CaCO3	N/A	[72]
	Nanoparticle Adsorption	biotinylated IL-6R protein	Avidin *	AuNP	BSA	[73]
	π–π Stacking (SAM)	MMP-9	MMP-9 antibody	Linker (PBASE)	BSA and ETA	[49]
		SNP	pDNA	Linker (PBASE)	ETA	[51]
	π–π Stacking	IL-6 protein (lysozyme)	PTDA	Direct (PTDA) with applied a negative electric field	N/A	[48]
		1X PBS	VR11 aptamer *	Linker (PBASE) and Direct (pyrene-tagged VR11)	Tween 20 solution	[52]
		Lysozyme protein from chicken egg white	amino linker modified anti-lysozyme DNA oligonucleotide	Linker (PBASE)	Tween 20	[59]
		DNT	TPET	rGO-TPET	N/A	[63]
		peptide (NT-proBNP)	aptamer	Linker (PBASE) with negative potential applied	ETA and BSA	[74]
		TNF-α protein	VR11	Linker (PBASE)	ETA and Tween 20	[75]
		SARS-CoV-2 NP, CRP, and S1-IgG and S1-IgM	CRP- and SARS-CoV-2-specific IgG assay configurations	Linker (PBA)	BSA	[76]

* VR11 aptamer: A single-stranded DNA aptamer selected for its high affinity and specificity to vascular endothelial growth factor receptor 1 (VEGFR-1), commonly used in biosensing applications. * Avidin: a biotin-binding protein commonly used in biosensors for signal amplification or conjugation strategies.

**Table 2:** Graphene-Based Biosensing Mechanisms.

Biosensor Type	Detection Mechanism	Application	Graphene Used	Detection Metrics	Ref.
** Optical Biosensors **	Surface Plasmon Resonance	Detect dengue virus e-proteins	rGO-PAMAM dendrimer	Sensitivity of 0.08–0.5 pM	[71]
	Photoluminescence	Demonstration of reversible charge transfer in the IR-780 iodide-MGF system	MGF	N/A	[18]
		Simultaneous detection of trace As^3+^, Cd^2+^, and Pb^2+^	GOQD	Achieved LoDs of 5.03 nM, 41.1 nM, and 4.44 nM	[84]
		Prostate cancer detection using PCA3 DNA probe	Au-GQD	LoD of up to 211 fM with 5 min response time	[85]
	Raman Spectroscopy-based	Label-free SERS detection of serum bilirubin for jaundice screening	GO-plasmonic gold nanostar	LoD of 0.436 μM	[87]
		Metal-free SERS platform for Rhodamine B sensing	Nitrogen-doped rGO	Enhanced Raman signal by the 103 order and LoD in micromolar (~10^−6^ M)	[88]
** Piezoelectric Biosensors **	Mass-sensitive	Wearable piezoelectric bio-MEMS device for detecting SARS-CoV-2 droplets	N/A	LoD of up to 79 ng/mL with 8 min response time	[24]
		Dual-mode gas sensor for TMA detection	NGO	LoD of 0.38 ppm with dual-signal QCM response	[139]
	Strain-sensitive	Wearable biosensor for human motion and swallowing detection	Graphene nanoparticles-doped PVDF fibre	4.56 V output at 5 wt% graphene	[58]
** Electrochemical (EC) Biosensors **	Impedimetric	Multiplexed glioblastoma DNA biomarkers detector	Graphene in DC and AC modes	LoD of 1 aM	[97]
		Portable impedimetric biosensor for SARS-CoV-2 N-protein detection	Graphene on PEDOT: PSS screen-printed strip	LoD of 56 fg/mL with range up to 10,000 pg/mL	[101]
		Bioimpedance tattoo sensor for continuous blood pressure monitoring	Graphene electronic tattoos	Grade A accuracy: SBP 0.2 ± 5.8 mmHg; DBP 0.2 ± 4.5 mmHg	[102]
	Amperometric	Urea monitoring using a urease/TrGO-based amperometric biosensor	Urease-functionalised TrGO	Sensitivity of 2.3 ± 0.1 μA cm^−2^ mM^−1^	[103]
		Enzymatic glucose biosensor	GOx/Fc-functionalised laser-induced graphene for glucose sensing	LoD of 0.04 µM with range of 0-11 mM and sensitivity of 11.3 µA mM^−1^ cm^−2^	[105]
		Non-enzymatic hydrogen peroxide (H_2_O_2_) sensing	3D GFs synthesized from glucose	LoD of 0.032 ± 0.005 μM	[106]
	Voltammetric	Label-free multiplexed immunosensor for precision breast cancer detection	MB-Chi/Gr/ITO electrodes	LoD of 0.04 pg/mL (CEA) and 0.04 mU/mL (CA153 and CA125)	[27]
		Label-free multiplexed detection of clinically relevant breast cancer microRNAs	AuNPs/GQDs/GO modified three-screen-printed carbon electrode (3SPCE) array	LoD of 0.04 fM (miRNA-21), 0.33 fM (miRNA-155), and 0.28 fM (miRNA-210)	[70]
		Multiplexed detection of Hg(II) and Cr(VI) using covalently dual-functionalised graphene	T-GO-C	Minimum LoD estimated at 1 ppb (Hg(II)) and 20 ppb (Cr(VI))	[68]
		Saliva-based biosensing of SARS-CoV-2 RNA	Lysozyme-dispersed rGO	200 ng/µL produced a ±25 µA current	[94]
		Non-enzymatic creatinine sensor	AuPs on a G-PLA 3D-printed electrode	LoD of 0.016 mmol/L	[107]
		Biosensor for SARS-CoV-2 cDNA		LoD of 0.30 µmol/L with sensitivity of 0.583 µA µmol^−1^ L	
		Multiplexed aptasensor for AMI biomarkers	Nanostructured gold-modified LSG	LoD of 1.65 ng/mL (cTnT), 2.58 ng/mL (cTnI), and 1.84 ng/mL (CRP)	[109]
		Portable rabies virus detector in bats using nasopharyngeal swab samples	rGO	LoD of 0.104 ng/µL with sensitivity of 0.321 µA (ng/µL)^−1^	[110]
** Transistor Biosensors (e.g., GFETs) **	Dirac Point	Aptamer-immobilised GFET for IL-6 biomarker detection	PTDA on a GFET	LoD of 100 pM	[48]
		VS-PEI nanoscaffold immobilisation of SARS-CoV-2 spike protein and human ferritin on GFET	Coplanar gated rGO FET	LoD of 0.74 nM (SARS-CoV-2 spike protein) and 0.23 nM (human ferritin)	[54]
		Label-free lysozyme protein sensor	Liquid-gated CVD-grown graphene FET	Concentration range at 10 nM to 1 uM	[59]
		Portable grapevine varietal detector	In-plane receded gated graphene FET	LoD of ~0.19aM	[93]
		Integrated ELISA protocol on a GFET for portable biosensing of ferritin	Coplanar gated rGO FET	Concentration range at 0.05 to 10 nM	[115]
		Microfluidic-GFET platform for detecting thrombin biomarkers	In-plane gated GFET array	LoD of 2.6 pM	[117]

**Table 3:** Multiplex Detection Strategies.

Multiplex Detection	Configuration	Application	Graphene Integration	Main Insights	Ref.
** Single Graphene–Single Bioreceptor **	Three graphene electrodes functionalised individually	Multiplexed aptasensor for acute myocardial infarction (AMI) biomarkers	Nanostructured gold-modified LSG	LoD of 1.65 ng/mL (cTnT), 2.58 ng/mL (cTnI), and 1.84 ng/mL (CRP)	[109]
	20 GFETs in groups of five	Virus proteins (*SARS-CoV-2*, RSV, Influenza A) and caffeine detection in wastewater	GFETs in PDMS wells with individual coplanar side gates	LoD of 55 ag/mL (SARS-CoV-2 spike protein), 408 ag/mL (Flu A), 453 ag/mL (RSV), and 26 fg/mL (on Caff209)	[125]
	12 coplanar liquid-gated GFET array with two common-source	Mathematical modelling of a liquid-gated GFET	Graphenea S-20 chip	Schwan’s Dispersion Theory Combined with Electron–Hole Puddle Theory for Graphene	[130]
	4-plex chip	Dual-mode GFET biosensor for *SARS-CoV-2* antigen and RNA detection	Five-channel GFETs	LoD of 103 PFU mL^−1^ (buffer), 104 PFU mL^−1^ (saliva), and ~65 aM (amplification-free viral RNA isolate)	[131]
** Single Graphene–Multiple Bioreceptors **	Covalently dual-functionalised Hg(II) and Cr(VI) on graphene electrode	Multiplexed metal ion detection	T-GO-C	Minimum LoD of 1 ppb for Hg(II) and 20 ppb for Cr(VI), with no cross-reactivity due to specific potential requirements	[68]
	Spatially functionalised paper substrate	μPAD-based chemiluminescence (CL) assay for multiplex detection of AIV biomarkers	N/A	LoD of 0.32 pM (H1N1), 0.34 pM (H7N9), and 0.29 pM (H5N1)	[72]
	Multiplexed fluorescence assay	Using distinct peptide substrates tagged with specific fluorescent dyes	Multicolor GO nanosensor	Protein kinase detection with LoD of 0.005 U/mL (A), 0.02 U/mL (Ab1), and 0.05 U/mL(Src)	[133]
	Eight electrical variables analysed with PCA	Multigas identification by analysing multiple electrical parameters of CNT-FET sensors	Pd/CNTs FET-type gas sensor	Operating concentration of 1 ppm (NO2, SO, CO, and H2S),10 ppm (H2), and 200 ppm (NH3)	[135]

## Data Availability

Data sharing is not applicable.

## Abbreviations

The following abbreviations are used in this manuscript:

ADC	Analogue-to-digital Converter
AIV	Avian Influenza Virus
AMI	Acute Myocardial Infarction
APTES	(3-Aminopropyl) triethoxysilane
AuNPs	Gold Nanoparticles
AuPs	Gold Particles
BSA	Bovine Serum Albumin
CESA	Cyclic Enzymatic Signal Amplification
CHA	Catalytic Hairpin Assembly
CL	Chemiluminescence
CNT	Carbon Nanotubes
CNT-FET	CNT field-effect transistor
COOH	Carboxyl Group
Cr(VI)	Chromium(VI)
CRP	C-Reactive Protein
CSV	Cell-Surface Vimentin
CTC	Circulating Tumour Cell
CTM	Circulating Tumour Microemboli
cTnI	Cardiac Troponin I
cTnT	Cardiac troponin T
CVD	Chemical Vapour Deposition
CV	Cyclic voltammetry
CNP	Charge Neutrality Point
DBCO	Dibenzocyclooctyne
DCM	Dichloromethane
DENV	Dengue virus
DPV	Differential Pulse Voltammetry
DNA	Deoxyribonucleic acid
DNT	2,4-dinitrotoluene
DSU	Dithiobis(succinimidyl undecanoate)
DVS	Divinyl Sulfone
EC	Electrochemical
EDC/NHS	1-Ethyl-3-(3-dimethylaminopropyl)carbodiimide/N-hydroxysuccinimide
EIS	Electrochemical Impedance Spectroscopy
ELISA	Enzyme-Linked Immunosorbent Assay
EpCAM	Epithelial Cell Adhesion Molecule
enPSERS	Enhanced Plasmonic SERS
ETA	Ethanolamine
FAM	6-Carboxyfluorescein
FETs	Field-Effect Transistors
FRET	Fluorescence Resonance Energy Transfer
FTH1	Ferritin Heavy Chain 1
FVIII	Factor VIII
G-PLA	Graphene-polylactic acid
GEMS	Graphene Electronic Multiplexed Sensor
GERS	Graphene-Enhanced Raman Scattering
GET	Graphene Electronic Tattoo
GFET	Graphene Field-Effect Transistor
GNR	Graphene nanoribbons
GNS	Gold nanostar
GNW	Graphene Nanowalls
GO	Graphene Oxide
GOQD	Graphene Oxide Quantum Dots
GQDs	Graphene Quantum Dots
Gr	Graphene
H_2_	Hydrogen
H_2_S	Hydrogen Sulphide
Hg(II)	Mercury(II)
IgG	Immunoglobulin G
IgM	Immunoglobulin M
IL-6R	Interleukin-6 Receptor
ITO	Indium Tin Oxide
LIG	Laser-induced graphene
LoD	Limit of Detection
LSG	Laser-Scribed Graphene
LSV	Linear Sweep Voltammetry
mAb	Monoclonal Antibody
MB-Chi	Methylene Blue-Chitosan
MCU	Microcontroller Unit
MGF	Monolayer graphene film
MI	Myocardial Infarction
MEMS/NEMS	Micro- and Nano-Electro-Mechanical Systems
MMP-9	Matrix Metallopeptidase 9
NGO	Nanoporous Graphene Oxide
NH_2_	Amino Group
NH_3_	Ammonia
NO_2_	Nitrogen dioxide
NP	Nucleocapsid protein
NPC	Nasopharyngeal Carcinoma
nf-EIS	non-faradaic Electrochemical Impedance Spectroscopy
PAD	Paper-Based Analytical Device
PAMAM	Polyamidoamine
PBA	1-Pyrenebutyric acid
PBASE	1-Pyrenebutyric acid N-hydroxysuccinimide ester
PBS	Phosphate-Buffered Saline
PCC	Protein-Catalysed Capture
PCA	Principal Component Analysis
PCA3	Prostate Cancer Antigen 3
Pd	Palladium
PDMS	polydimethylsiloxane
pDNA	Plasmid deoxyribonucleic acid
PE	Pulmonary Embolism
PEDOT:PSS	poly(3,4- ethylenedioxythiophene):poly(styrene sulfonate)
PEG4	Polyethylene Glycol (with 4 repeating units)
PEI	Polyethyleneimine
PTDA	Pyrene-Tagged DNA Aptamer
PVDF	Polyvinylidene fluoride
PL	Photoluminescence
POC	Point-of-care
QCM	Quartz Crystal Microbalance
rGO	Reduced Graphene Oxide
RABV	Rabies Virus
RhB	Rhodamine B
RNA	Ribonucleic Acid
RSV	Respiratory Syncytial Virus
SAM	Self-Assembled Monolayer
SARS-CoV	Severe Acute Respiratory Syndrome Coronavirus
SERS	Surface-Enhanced Raman Spectroscopy
SNP	Single-Nucleotide Polymorphism
SO_2_	Sulphur dioxide
SPR	Surface Plasmon Resonance
SWV	Square Wave Voltammetry
T-GO-C	Thymine-Graphene Oxide-Carbohydrazide
TMA	Trimethylamine
TNF-α	Tumour Necrosis Factor alpha
TPET	Tetraphenylethylene Pyrene
TPU	Thermoplastic Polyurethane
TrGO	Thermally Reduced Graphene Oxide
VOC	Volatile Organic Compound
VS-PEI	Vinylsulfonated-polyethyleneimine
XPS	X-ray Photoelectron Spectroscopy
